# Nematic superconducting state in iron pnictide superconductors

**DOI:** 10.1038/s41467-017-02016-y

**Published:** 2017-12-01

**Authors:** Jun Li, Paulo J. Pereira, Jie Yuan, Yang-Yang Lv, Mei-Ping Jiang, Dachuan Lu, Zi-Quan Lin, Yong-Jie Liu, Jun-Feng Wang, Liang Li, Xiaoxing Ke, Gustaaf Van Tendeloo, Meng-Yue Li, Hai-Luke Feng, Takeshi Hatano, Hua-Bing Wang, Pei-Heng Wu, Kazunari Yamaura, Eiji Takayama-Muromachi, Johan Vanacken, Liviu F. Chibotaru, Victor V. Moshchalkov

**Affiliations:** 1 Research Institute of Superconductor Electronics, Nanjing University, 210093 Nanjing, China; 2 National Institute for Materials Science, Tsukuba, 305-0044 Japan; 3 INPAC-Institute for Nanoscale Physics and Chemistry, KU Leuven, B-3001 Leuven, Belgium; 4 Theory of Nanomaterials Group, KU Leuven, B-3001 Leuven, Belgium; 5 National Laboratory for Superconductivity, Institute of Physics, and Beijing National Laboratory for Condensed Matter Physics, Chinese Academy of Sciences, 100080 Beijing, China; 6 Wuhan National High Magnetic Field Center and School of Physics, Huazhong University of Science and Technology, 430074 Wuhan, China; 7 Electron Microscopy for Materials Research (EMAT), University of Antwerp, B-2020 Antwerp, Belgium; 8 Synergetic Innovation Center in Quantum Information and Quantum Physics, University of Science and Technology of China, Hefei, 230026 Anhui China; 9 Graduate School of Chemical Sciences and Engineering, Hokkaido University, Sapporo, 060-0810 Japan

## Abstract

Nematic order often breaks the tetragonal symmetry of iron-based superconductors. It arises from regular structural transition or electronic instability in the normal phase. Here, we report the observation of a nematic superconducting state, by measuring the angular dependence of the in-plane and out-of-plane magnetoresistivity of Ba_0.5_K_0.5_Fe_2_As_2_ single crystals. We find large twofold oscillations in the vicinity of the superconducting transition, when the direction of applied magnetic field is rotated within the basal plane. To avoid the influences from sample geometry or current flow direction, the sample was designed as Corbino-shape for in-plane and mesa-shape for out-of-plane measurements. Theoretical analysis shows that the nematic superconductivity arises from the weak mixture of the quasi-degenerate *s*-wave and *d*-wave components of the superconducting condensate, most probably induced by a weak anisotropy of stresses inherent to single crystals.

## Abstract (web summary)

Nematic electronic order is rare and its onset often indicates a phase transition. Here, Li et al. report a nematic superconducting state in Ba_0.5_K_0.5_Fe_2_As_2_ by measuring the angular dependence of the in-plane and out-of-plane magnetoresistivity.

## Introduction

The discovery of iron-based superconductors with high critical temperature has revived the interest for unconventional superconductivity^1^. Their similarity to high-*T*_c_ cuprates^2^ suggests that magnetic fluctuations might be the leading mechanism of superconducting pairing^3, 4^. The peculiar feature of these materials is the essentially multiband character of pairing^4–6^. The Fermi surface of iron-based superconductors is very rich and can modified by introducing doping atoms^5, 7, 8^, which mostly change the number of electrons in the bands near the Fermi level. Moreoer, the symmetry of the electron pairing appears to be intertwined with the topology of the Fermi surface which varies drastically across the compounds^5, 7, 8^. Furthermore, a crossover between different pairing types, namely, between *s*_±_-wave and *d*-wave symmetry, under variation of electron/hole doping is highly debated in the community^9–11^, however, consensus is not reached with several opposing parties^12–14^. At the same time, other authors claim the near-degeneracy of *s*- and *d*-wave components^15, 16^ or strong subdominat *d*-wave component^17^ in iron-based superconductors, particularly, in Ba_1−*x*_K_*x*_Fe_2_As_2_^18^.

Iron-based superconductors display an antiferromagnetic dome in the temperature-doping phase diagram, centered in the parent compound^19^. Numerous studies revealed the existence of nematic order just above the magnetic ordering phase, characterized by the tetragonal rotational symmetry breaking and always accompanied by the structural transition to orthorhombic phase^19–22^. The superconducting state nucleating at the bottom of this dome in electron- or hole-doped compounds displays nematic symmetry as well, which is testified, e.g., by the C_2_ symmetry of the superconducting gap along the Fe–Fe bond direction^23^. However, it should be mentioned that the magnetic order (no pressure) is absent in the FeSe system, and the nematic state also exists in the normal state, further researches are necessary to understand the mechanism of the nematic state in superconductivity. Electronic nematic orders are ubiquitous also in other unconventional superconductors, such as high-*T*_c_ cuprates and heavy-fermion compounds^24^. They always nucleate in the normal phase, most probably via spin-fluctuation mechanism^19^, and depend on the degree of doping. Thus in hole-doped iron-based superconductors such as Ba_1−*x*_K_*x*_Fe_2_As_2_ the nematic order is observed till *x* ≈ 0.3, when the antiferromagnetic phase boundary is reached, and disappears at higher values of doping including the optimal one^25^ (*x* = 0.4).

Here we present evidence of a nematic superconducting state which is not accompanied by tetragonal symmetry breaking of the lattice or onset of magnetic order. Measuring angular dependences of the in-plane magnetoresistivity in single-crystalline samples of iron-based superconductors near optimal hole doping, we find twofold oscillations with the rotation of the applied magnetic field. The C_2_ symmetry of the superconducting condensate persists from the nucleation till low temperatures and for a range of dopings near *x = *0.5. The reason for this unusual nematic state is found to be a mixture of quasi-degenerate *s*_±_-wave and *d*-wave components of the superconducting condensate by weak anisotropy of stresses and defects distribution in single-crystalline films.

## Results

### Measurement setup

**Fig. 1:**
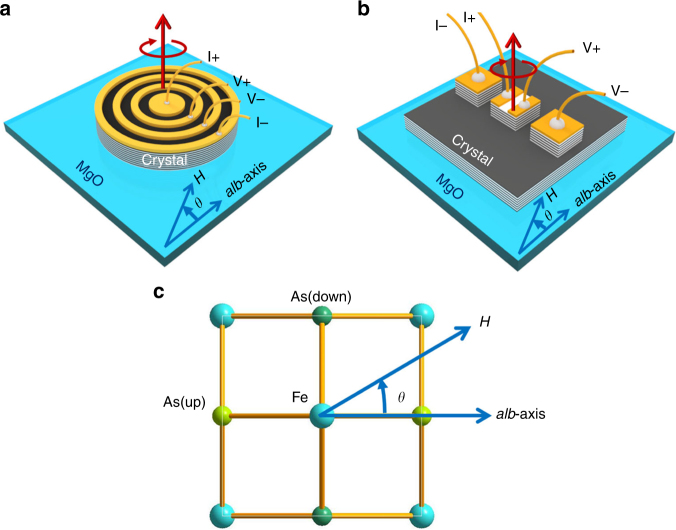
Schematic image of the sample geometry. **a** Diagram of the Corbino-shape device for angular-dependent IMR *ρ*_*ab*_ measurements. The electric current is lead to flow radially from the center to the outermost electrode, where the outermost electrode was in diameter of 80 μm and the detailed sample geometry is given in Supplementary Fig. 2. The magnetic field was applied to the *ab*-plane with an angular error of less than ±1°. The angle was set to zero (*θ* = 0) when the field was parallel to one of *a*(*b*)-axis. Then the sample was rotated within the *ab*-plane to tune *θ* between *H* and the *a*(*b*)-axis. **b** Diagram of the mesa device for angular-dependent IMR *ρ*_*c*_ measurements, where the thickness of the mesa was 1.5 μm (see sample geometry in Supplementary Fig. 4). The magnetic field was applied within the *ab*-plane. **c** Schematic image of the rotating crystal to adjust the angle *θ* between *H* and the *a*(*b*)-axis as well

The optimally doped single crystals of Ba_0.5_K_0.5_Fe_2_As_2_ with a *T*_c_ = 38 K were grown by a high-pressure method^26^. An annular-electrode method was adopted to measure the in-plane electrical resistivity (*ρ*_*ab*_) of selected single crystal (Supplementary Fig. 2); the outermost annular golden pattern was fabricated with a diameter of 80 μm (Fig. 1). The electric current flows radially from the center to the outermost electrode as in the Corbino disc^27^, thus eliminating the anisotropic Lorentz force effect. The *ρ*_*ab*_ was measured with the Physical Properties Measurement System (PPMS; Quantum Design). The angle *θ* is defined as that between the magnetic field and the *a*(*b*)-axis of the lattice, as indicated in Fig. 1a. The dependence of *ρ*_*ab*_ on *θ* was measured by rotating the *ab*-plane around the *c*-axis in a fixed magnetic field parallel to the *ab*-plane and at a fixed temperature. The potential misalignment of the magnetic field against the *ab*-plane was estimated to be less than ±1° (Supplementary Fig. 11). Variation of the sample temperature due to sample rotation was verified to be less than 0.008 K (Supplementary Fig. 12).

### In-plane magnetoresistivity

**Fig. 2:**
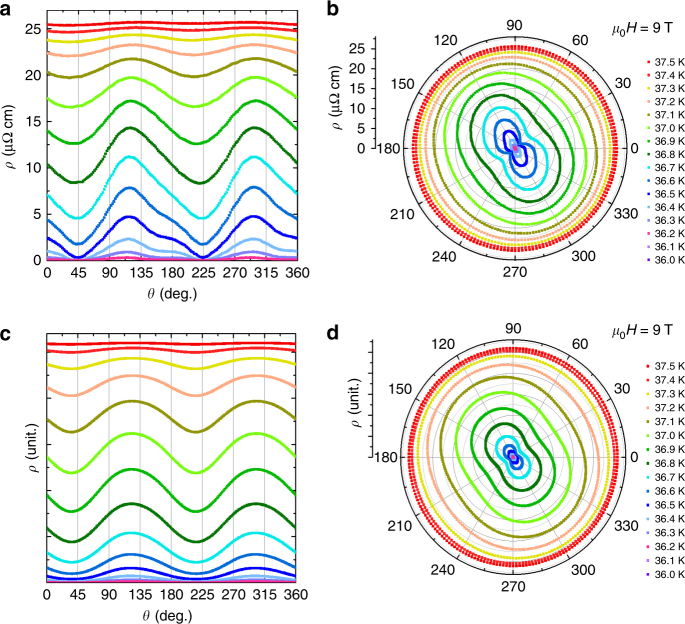
Experimental and theoretical angular dependence IMR. Experimental (**a**) and theoretical (**c**) values of the angular dependence of the IMR, and respective polar plots of IMR experimental (**b**) and theoretical (**d**) values, at various temperatures for the applied magnetic field of 9 T for which the experimental values were obtained using the Corbino disk measurement configuration. Theoretical values correspond to the model with three components with the symmetries *s*_±_, $d_{x^2 - y^2}$ and *d*_*xy*_

**Fig. 3:**
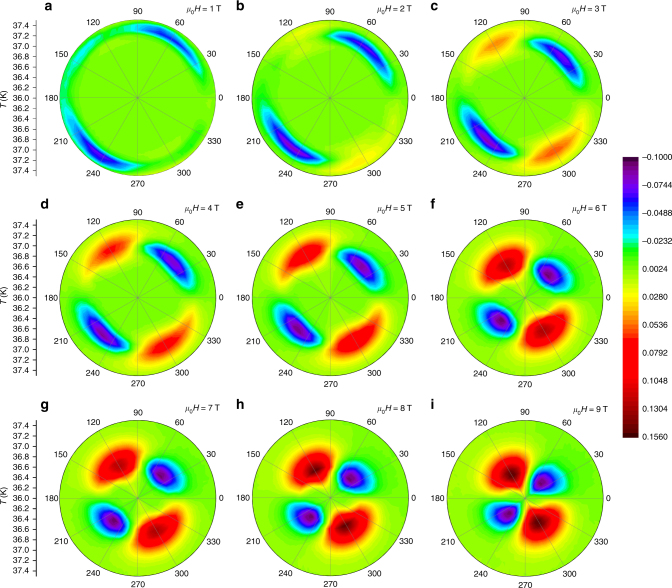
Color contours of normalized magnetoresistivity. Angle-dependent normalized magnetoresistivity (*ρ* − *ρ*_0_)/*ρ*_*n*_ at various temperatures and magnetic fields. The color bar represents the normalized magnetoresistivity (*ρ* − *ρ*_0_)/*ρ*_*n*_, where *ρ* is *ρ*_*ab*_, *ρ*_0_ is the *ρ*_*ab*_ at *θ* = 0, and *ρ*_*n*_ is the normal state *ρ*_*ab*_ at a temperature of 39 K

Figure 2a and b shows the measured angular dependence of the in-plane magnetoresistivity *ρ*_*ab*_ (IMR) in vicinity of superconducting transition under magnetic field of 9 T. The *ρ*_*ab*_ displays oscillations as a function of *θ* below the *T*_c_ -onset. These oscillations disappear as the temperature approaches *T*_c_-onset from below, reaching an isotropic resistivity. Away from *T*_c_-onset and closer to the *T*_c_-offset, a twofold oscillation of IMR is observed as a nearly sinusoidal variation, in which the maxima of IMR appears at *θ* ≈ 135° and 315°, and the minima at *θ* ≈ 45° and 225°. Figure 3 shows the change in symmetry under various magnetic fields and temperatures more clearly. The color contours represent the normalized magnetoresistivity (*ρ* – *ρ*_0_)/*ρ*_n_, where *ρ* is *ρ*_*ab*_, *ρ*_0_ is the *ρ*_*ab*_ at *θ* = 0, and *ρ*_*n*_ is the normal state *ρ*_*ab*_ at a temperature of 39 K. Note that the anisotropic factor can be up to −10 to 15.6% for the minima and maxima magnetoresistivity, respectively. Such anisotropic factors are markedly larger than that of the structure distortion induced anisotropic one, which is normally around 0.1% along the *a*- and *b*-axis.

We carried out *ρ*_*ab*_ measurements for more than 10 different single crystals and found that the maximum (minimum) of *ρ*_*ab*_(*θ*) is always in the close neighborhood to the (±1, ±1, 0) direction of the crystal, namely, the Fe–Fe (ΓM) bond direction. To confirm that the *ρ*_*ab*_(*θ*) maxima (minima) are independent of the initial direction of the applied magnetic field, similar measurements were repeated at different initial angle *ϕ* of 45° and 90° (Supplementary Fig. 13). The maximum (minimum) of *ρ*_*ab*_(*θ*) was always observed along the direction of the Fe–Fe bond. When the temperature is close to the *T*_c_-offset, the *ρ*_*ab*_ shows a mixture of twofold and fourfold oscillations (see Supplementary Figs. 9 and 10). The fourfold oscillation, however, has a considerably lower amplitude.

Our central finding here is that the IMR indicates a twofold oscillation as a function of *θ* when the temperature is set near the mid-point of the superconducting transition. If the temperature is much higher than the mid-point, the oscillation disappears with a constant resistivity independent of *θ*. When the temperature is much lower than the mid-point, on the contrary, the resistivity becomes zero for the whole range of *θ*. Increase of the magnetic field does not affect the phase of the oscillation but makes its amplitude larger.

### Out-of-plane magnetoresistivity

Complementally, *ρ*_*c*_(*θ*) was measured by using the mesa technique (well-studied on the cuprates superconductors^28^). Figure 1b gives the scheme of the mesa structure in which the current flows from the top side of the mesa to the crystal substrate. *μ*_0_ *H* was applied within the *ab*-plane and the sample was rotated around the *c*-axis as in the IMR experiments. Thus the Lorentz force effect is eliminated as previously, because *μ*_0_ *H* is always perpendicular to the current flow direction. The angular-dependent *ρ*_*c*_ in the superconducting transition region is given in Supplementary Fig. 6. We can see that *ρ*_*c*_(*θ*) demonstrates oscillations similar to *ρ*_*ab*_(*θ*).

### In-plane upper critical field

**Fig. 4:**
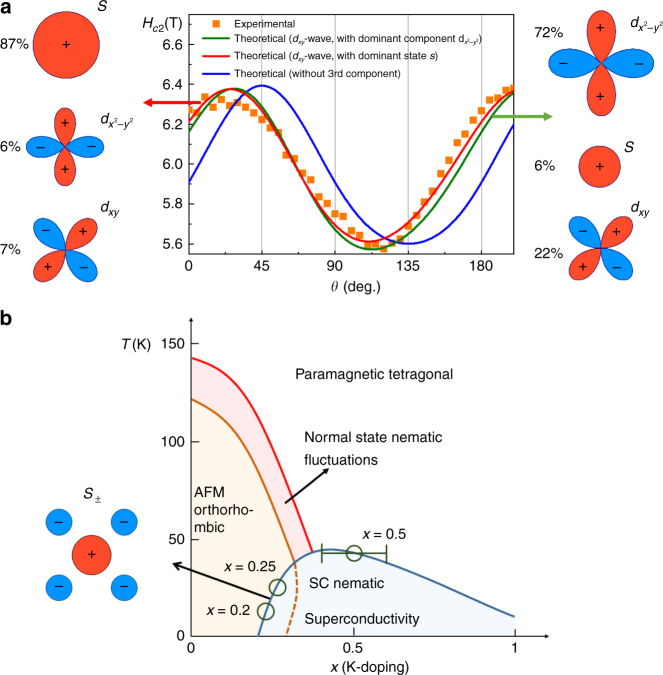
Upper critical fields and phase diagram. **a** Angular dependence of the second magnetic critical field at 38.4 K (*T*_c_ ≈ 39 K), retrieved from transport experiments (black filled square symbol) in the Corbino disc measurement configuration, from theoretical model with *s*-wave and $d_{x^2 - y^2}$-wave symmetries (full *blue* line), from theoretical model with *s*_±_-wave, $d_{x^2 - y^2}$-wave and *d*_*xy*_-wave symmetries (full red and blue lines depending on dominant component). The mixing of the different symmetry components of the order parameters is indicated on the left and right side panels next to a schematic representation of each component of the order parameter as function of the internal momentum of the Cooper pairs. The indicated percentages correspond to the relative weights (*r*_1_, *r*_2_, and *r*_3_) of the wave function coefficients, ${\mathrm{\Phi }}\left( {\bf{k}} \right) = r_1{\mathrm{\Phi }}_s\left( {\bf{k}} \right) + r_2{\mathrm{\Phi }}_{d_{x^2 - y^2}}\left( {\bf{k}} \right) + r_3{\mathrm{\Phi }}_{d_{xy}}\left( {\bf{k}} \right)$. In this schematic representation red and green indicate positive and negative value of the components, respectively. **b** Phase diagram of hole-doped Ba_1−*x*_K_*x*_Fe_2_As_2_ iron-based superconductors. There is controversial evidence concerning the symmetry of the order parameter in the doping domain close to *x* = 1: refs. ^12–14^ argue in favor of *s*_±_ and ref. ^11^ (and references therein) in favor of $d_{x^2 - y^2}$ pairing symmetry. The error bar for *x* = 0.5 corresponds to 15 samples with *x* ranging from 0.45 to 0.55. The red region demonstrates the normal state nematic fluctuations, which may originate from magnetic order, structural or charge/orbit order transition^19^. The superconducting (SC) nematic state is observed on the basis of the present IMR results (Figs. 2 and 3)

Magnetoresistivity can be linked to thermally activated vortex creep and flux flow at the offset and the onset of critical temperature, respectively, therefore an anisotropy of the pinning potential might be the reason for the observed C_2_ oscillations of IMR^29^. To check this possibility, we extracted the second critical field *H*_*c*2_, which does not depend on the pinning potential, from electrical transport measurements. Figure 4a shows that *H*_*c*2_(*θ*) displays the same anisotropic behavior, thus, excluding anisotropic distribution of the pinning sites as the source of the *ρ*_*ab*_(*θ*) tetragonal symmetry breaking. We also employed the pulsed high magnetic fields (57 T) to study on the *H*_*c*2_(*θ*) in relatively low temperature of 35 K (4.5 K below the *T*_*c*_), similar twofold anisotropic behavior (see Supplementary Figs. 7–9) was found as those of static magnetic fields.

The procedure used here allows to measure *ρ*_*ab*_ with high accuracy and low noise, however, it might induce metastable states when the field is deviated from the initial direction. To rule out this possibility, field-cooling experiments were made in which the fixed magnetic field was applied at *T* = 140 K, whereas the temperature was lowered till the measuring value, the procedure being repeated for each direction of the applied field. The obtained angular dependencies of IMR are practically identical to the ones obtained with the previous procedure (see Supplementary Note 8), indicating the absence of metastability in our measurements.

### Technical error exclusion

Analyzing possible reasons for tetragonal symmetry breaking of *ρ*_*ab*_(*θ*), *ρ*_*c*_(*θ*), and *H*_*c*2_(*θ*), we note that according to the diagram established for bulk Ba_1−*x*_K_*x*_Fe_2_As_2_^30^, the present materials (*x* ≈ 0.5) are expected to be free of orthorhombic distortions or antiferromagnetic ordering. Tunneling electronic microscopy (TEM) measurements also show the absence of twinning till low temperatures (Supplementary Fig. 1). This implies that symmetry breaking mechanisms in the normal phase, like orbital and spin instabilities, can be ruled out in the present case. To rule out effect of shape, samples of different geometries, including those of circular shapes, were additionally investigated and have shown no qualitative differences for the angular dependence of IMR. Finally, we investigated the effect of misalignment of the *c*-axis of the crystal and the rotation axis on the amplitude of the IMR oscillation, and found that it is several orders of magnitude smaller than the observed ones (see the Supplementary Fig. 11).

To summarize, the geometry of the samples and of the setup, as well as the anisotropy of vortex pinning potential and the metastability can be ruled out as possible causes for the C_2_ anisotropy of *ρ*_*ab*_(*θ*) and *ρ*_*c*_(*θ*). The lattice remains tetragonal for the optimally doped crystal from room to low temperatures and no magnetic ordering is observed^30^, which excludes usual mechanisms such as orthorhombic structural phase transition, orbital and spin instabilities as reasons for prominent twofold symmetry of IMR in Fig. 2. In addition, in all measurements the anisotropy of IMR can only be observed in the transition domain between the normal and superconducting state. This experimental analysis demonstrates that the observed tetragonal symmetry breaking is truly unusual, and is intrinsically intertwined with the superconducting state. Contrary to conventional nematic states reported for tetragonal superconductors^8, 19–24^, we face here a new manifestation of nematicity, the nematic superconducting state.

## Discussion

The observed nematicity in the superconducting state could in principle arise from spontaneous symmetry breaking in the coupling between superconducting and nematic order parameter^9^. This would naturally explain why the nematic order is absent in the normal phase and only shows up in superconducting state. However, on symmetry reasons the nematic order parameter *φ* can only couple simultaneously to two superconducting order parameters of different symmetry, Δ_*s*_ and Δ_*d*_, in a fashion ∝*φ*Δ_*s*_Δ_*d*_^9^, which leads to its equilibrium value of the form *φ* ^(0)^ ∝ Δ_*s*_Δ_*d*_. The latter is quadratic in superconducting order parameter and, therefore, negligible at the nucleation point. This contradicts *H*_*c*2_(*θ*) data (Fig. 4a), which are consistent with the existence of nematic superconductivity already at the nucleation. Thus, a simple Ginzburg–Landau argument allows to rule out the spontaneous symmetry breaking as possible reason for the observed large C_2_ oscillations of IMR and *H*_*c*2_(*θ*).

We are forced, therefore, to assume that the observed nematicity of the superconducting state is triggered by tetragonal symmetry breaking present already in the normal phase, despite the lack of its evidence from structural data. To prove the existence of the tiny nematicity in the normal state, we digitalized IMR data at *T* > *T*_c_ and found indeed their low-symmetry angular oscillation with an amplitude of 0.8% (Supplementary Fig. 16; Supplementary Table 1). This preexisting weak nematicity can induce significant tetragonal symmetry breaking in the superconducting state only in the presence of quasi-degenerate components of superconducting order parameters of different symmetry, which seems indeed to be the case for Ba_0.5_K_0.5_Fe_2_As_2_. The current understanding is that in the hole optimally doped and underdoped range (*x* ≤ 0.4), Ba_1−*x*_K_*x*_Fe_2_As_2_ is likely to exhibit *s*_±_-wave pairing^11^, whereas in the strongly hole-doped range it shows evidences of *d*-wave superconductivity, particularly for the completely doped, namely, KFe_2_As_2_^11, 31, 32^. At intermediate doping levels, a crossover between these two pairing symmetries is expected^10^. On the basis of these facts, we performed microscopic calculations of *H*_*c*2_(*θ*) taking into account possible mixing of *s*_±_-wave and *d*-wave superconducting components. In these calculations, we assumed that the samples are close to the clean limit due to a very short coherence length (~3 nm) and the existence of strong angular oscillations of the IMR and the critical field.

*H*_*c*2_(*θ*) was calculated using the linearized Eilenberger equations, suitable to treat the clean limit. The Fermi surface and the Fermi velocity were obtained within the five-band model^18^ in the rigid band approximation (see Supplementary Notes 1–4 for more details). The pairing potential was parametrized by a procedure similar to refs ^4, 33^. Magnetoresistivity in the transition domain from normal to superconducting state was calculated using Tinkham’s model^34^.

The parametrization of the pairing potential includes only one free parameter, the critical temperature of the secondary component (*T*_c2_). Fixing it, the critical temperature of the main component (*T*_c1_) is found from the known critical temperature of the system (*T*_c_). As *T*_c2_ approaches *T*_c1_, the system becomes unstable under small perturbations that mix the two components. By admixing a small term to the pairing potential, we obtained a *H*_*c*2_ and resistivity angular dependencies with similar features to the experimental curves, Figs. 2 and 4a, respectively. The maxima and the minima of the simulated *H*_*c*2_ and resistivity curves are along the Fe–Fe bond direction and a strong anisotropy is present despite the fact that only an arbitrary small admixed term is required to obtain this result. Similar results were obtained in the cases, *T*_c1_ > *T*_c2_ and *T*_c1_ < *T*_c2_. Importantly, the angle *θ* at which the maximum of IMR and the minimum of *H*_*c*2_ arises is only defined by the symmetry of the mixing components of superconducting order parameter, *s*_±_ and $d_{x^2 - y^2}$, and does not depend on the details of the weak symmetry breaking triggering the observed nematicity. It is also independent from the extent of mixing of the *s*_±_-wave and *d*-wave superconducting components. Finally, the simulation results depend weakly on changes in the Fermi surface and electron pockets eccentricity corresponding to small variations of the doping level.

The fact that the anisotropy direction of IMR and *H*_*c*2_(*θ*) varies weakly across the set of investigated samples, and that it is reproduced by a simple model for a wide range of its parameters, is the major evidence of the validity of the proposed mechanism for nematic superconducting state. The correct anisotropy angle is obtained exclusively from the admixture of the $d_{x^2 - y^2}$-wave component. An additional support for this scenario comes from a very small value of the mixing term in the pairing potential needed to reproduce the main features of angular dependence of IMR and *H*_*c*2_(*θ*). For example, the necessary extent of mixing of the two superconducting components whose critical temperatures (*T*_c1_ and *T*_c2_) differ by 2 K would require an amplitude of the mixing term in pairing potential amounting to only 1% of the main (tetragonal) pairing potential. This is in line with the experimental fact that the preexisting. As there is no evidence for nematic order in bulk compounds with optimally or over doped compositions^30^, we should admit that the weak tetragonal symmetry breaking observed here in the normal phase (Supplementary Fig. 16) is rooted in their preparation procedure of the single-crystalline films. Indeed, the latter have been grown under a very high pressure (~3 GPa)^26^, which explains the presence of anisotropic stresses in the *a*–*b* plane inducing at their turn, a weak tetragonal symmetry breaking. The extrinsic reason for the latter explains naturally (i) the weakness of the symmetry breaking in the normal phase and (ii) its uniform character required for the observability of the twofold oscillations of the IMR and *H*_*c*2_(*θ*) in the whole sample. Indeed, if the tetragonal symmetry would be broken spontaneously, domains with different nematic orientations should have appeared. As a result, the IMR and *H*_*c*2_(*θ*) anisotropy would have averaged to zero, leaving only fourfold oscillations. Finally, we can understand why the weak symmetry breaking in the normal phase is not detected as symmetry lowering of the lattice structure. For the sake of estimation one can relate the relative variations of the lattice constants *a* and *b* to the relative variations of the corresponding resistivity *ρ*_*a*_ and *ρ*_*b*_ (see, e.g., Fig. 4 in ref. ^33^). Then for angular oscillations of resistivity of 0.4% (Supplementary Fig. 16b), we obtain for orthorombicity (*a* − *b*)/(*a* + *b*), a value of ca 0.005%, apparently too small to be observed. On the same reason the tetragonal symmetry breaking will not be detected in the magnetic susceptibility as well.

To emphasize the difference between the observed nematic superconducting state and the superconductivity in the presence of conventional nematic order (arising from electronic/structural instability in the normal phase), we measured the angular-dependent IMR for underdoped single crystals Ba_0.75_K_0.25_Fe_2_As_2_ and Ba_0.8_K_0.2_Fe_2_As_2_, with doping values under the antiferromagnetic dome. The obtained angular-dependent IMR differs drastically from the one in Fig. 2 (see also in Supplementary Figs. 14 and 15). In contrast to the latter, it displays a twofold symmetry with minima and maxima aligned exactly along the *a*- and *b*-axis, respectively, following precisely the direction of orthorhombic structural distortion as expected.

Besides the described major features of *ρ*_*ab*_(*θ*) and *H*_*c*2_(*θ*), experimental results display two additional ones, which are not captured by the two-component model. First, there is a deviation of the angle of maximum of IMR (minimum of *H*_*c*2_(*θ*)) from the Fe–Fe bond direction and, second, there is a broken reflection symmetry across the line passing through the two maxima of IMR. We first tried to exhaust the possibilities by changing fitting parameters, relaxing constraints and approximations within the two-component model to explain these features. We also tried to improve our model, adding higher harmonics in the potential decomposition. Finally, we hypothesize that the additional symmetry breaking could be explained by admixing a third component of superconducting order parameter of a different symmetry from the other two. Note this is only a small fine-tuning of the main result, i.e. symmetry breaking mechanism arising from the mixture of *s*_±_- and $d_{x^2 - y^2}$-wave components responsible for the direction of IMR lobes close to 135°. Concerning the multiband character of superconductivity in these compounds, theory^16, 18^ and experiment^15, 17^ give controversial evidence about the subdominant superconducting components. Simulations of *ρ*_*ab*_(*θ*) and *H*_*c*2_(*θ*) within the three-component model give for critical temperatures of the components values spanning a temperature domain of ca ~10 K (see Supplementary Table 2). We considered two symmetries for the third admixed component, *d*_*xy*_ or *g*-wave, which gave similar results (Fig. 4b). Obviously, the admixture of the third component of the superconducting order parameter is supposed to occur on the same reason as the mixture of the first two.

Although the nematic superconducting state in the present compounds is ultimately induced by the tetragonal symmetry breaking in the normal phase, it differs qualitatively from ordinary nematicity observed in underdoped compounds in the orthorombic structural phase. For the latter, the extrema of IMR curves follow strictly the orthorombicity axes *a* and *b* in both normal and superconducting phases (Supplementary Figs. 14 and 15). This is a consequence of orthorombic Fermi surface leading to anisotropic (C_2_ symmetry) pairing potential and superconducting order parameter. On the contrary, the IMR lobes are found close to 135° in the superconducting phase of all compounds with *x* ≈ 0.5 investigated here, pointing to the fact that IMR remains qualitatively unchanged for any form of extrinsic symmetry breaking in the normal phase. This behavior can be basically explained by the mixing of tetragonal *s*_±_ and $d_{x^2 - y^2}$ order parameters in all investigated compounds. The extrinsic symmetry breaking inducing this mixing is expected to be very weak, as testified by an almost isotropic IMR in the normal phase (Fig. 16), thus having the role of a trigger rather than of a driving force for nematicity like in underdoped compounds.

## Methods

### Synthesis of crystals

Single crystals of Ba_1−*x*_K_*x*_Fe_2_As_2_ (*x* = 0.2, 0.25, and 0.5) were grown using a high-pressure method (~3 GPa)^26^. X-ray diffraction confirmed that they have the tetragonal lattice, consistent with previous reports. The compound takes the tetragonal lattice form over the entire temperature range above 2 K^30^. For the optimal-doped crystal with nominal content *x* = 0.5, although electron probe microanalysis revealed various potassium contents from 0.45 to 0.55 per formula unit, the crystals are still far from the orthorhombic and antiferromagnetic region, according to the well-established phase diagram^30^. Low temperature transmission electronic microscopy (TEM) measurements show the absence of intrinsic twin boundaries at temperature above 96 K (see Supplementary Fig. 1).

### Fabrication of Corbino samples

A selected single crystal was cleaved over the *ab*-plane with a thickness of 1 μm. An annular-electrode method was adopted to measure the in-plane electrical resistivity (*ρ*_*ab*_) (Fig. 1a); the outermost annular golden pattern was fabricated with a diameter of 80 μm (see Supplementary Fig. 2). To avoid the interface oxidation or degeneration between the crystal and conducting films, the samples were deposited on Au in an in-situ fabrication system for micro- and nano-device (AdNaNo-Tek Ltd.), where the samples were in inert (Ar) or high-vacuum condition (~10^−10^ Torr). The fabrication processes of Corbino disk are given as following: (a) The crystal was firstly cleaved into pieces with micrometers in thickness, by using scotch tape, and then glued on MgO substrate with the *ab*-plane parallel to the substrate surface using a thin layer of epoxy. After heat-treated the epoxy at 150 °C for 4 h, the crystals were further cleaved into 1 ~ 2 μm in thickness. To improve the electrical contact, a 120-nm Au layer was evaporated onto the sliced crystal (see Supplementary Fig. 2a). The samples were then heat-treated in vacuum at 300 °C for 24 h. (b) The crystal was fabricated into a disk shape using photolithography technique, after which Ar-ion milling was used to remove the crystals around the disk (see Supplementary Fig. 2b), and then the photoresist was removed using acetone. (c) Using another photolithography technique to pattern circuits on the disk, and Ar-ion-milling the Au layer into circle mesa in the middle of the disk (Supplementary Fig. 2c). (d) A 100-nm insulating SiO layer was then coated onto the crystal, and then remove the photoresist. Thus, the crystal was covered by the SiO layer except the Au circle mesas, here the SiO layer exhibits as dark blue as shown in Supplementary Fig. 2d. To avoid the possible shortage around the disk edge, we covered a thin layer of epoxy at edge region around the disk. (e) Half part of the Au circle mesas was covered by another layer of SiO using a lift-off technique as shown in red part in Supplementary Fig. 2e. The edge of the disk was coated with a thin layer of epoxy to avoid possible charge shortage. (f) The whole sample was deposited another layer of 120-nm Au layer, and the fabricated the electrodes for the annular mesa by using a further step of photolithography and argon-ion etching (see Supplementary Fig. 2f).

### Fabrication of mesa samples

The out-of-plane magnetoresistivity (*ρ*_c_) was measured by using the mesa technique on the crystal of Ba_0.5_K_0.5_Fe_2_As_2_ as shown in Fig. 1b. The cleaved single crystals were mounted on a MgO substrate and a 120-nm Au layer was evaporated on the crystal in the in-situ fabrication system. A series of mesas—~1.5 μm in thickness with various surfaces of 10 × 10, 20 × 20, and 40 × 40 μm^2^—was fabricated using a photolithography technique (see Supplementary Fig. 4). A 120-nm insulating SiO layer was coated to surround the mesa edges. The electrodes were contacted on the top of the mesas by additional photolithography processes. Supplementary Fig. 5 shows the temperature dependence of *ρ*_c_.

### Static fields measurements for angle-dependent magnetoresistivity

The static field measurements are carried out in the PPMS; Quantum Design. For the Cobino samples, the electric current flows radially from the center to the outermost electrode as in the Corbino disk as shown in Fig. 1, thus eliminating the Lorentz force effect. The *ρ*_ab_ was calculated by the equation *ρ*_ab_ = 2π*hR*/ln(*r*_2_/*r*_1_), where *h* is the thickness of the crystal, *R* is the resistance, and *r*_1_ and *r*_2_ are the radii of the two voltage terminals. Here, the static magnetic fields from 1 to 9 T are applied in PPMS.

### Pulsed high magnetic field experiments for angle-dependent magnetoresistivity

The pulsed high magnetic fields experiments are studied in Wuhan National High Magnetic Field Center and School of Physics. Here, we selected the sample as ultrathin micro-bridges Ba_0.5_K_0.5_Fe_2_As_2_. The fabrication process of the micro-bridge was introduced in ref. ^35^ The corresponding pulsed high magnetic fields experiment was carried out as shown in Supplementary Fig. 7. The sample was rotated along the *c*-axis, and the magnetic field up to 57 T was applied within the *ab*-plane.

### Data availability

All data generated or analyzed during this study are included in this published article (and its supplementary information files) or are available from the corresponding author upon request. The computer code generated during the current study is available from the corresponding author on request.

## Electronic supplementary material

Supplementary Information
