# Supplementary material for: Nematic superconducting state in iron pnictide superconductors
Source: Nat Commun. 2017 Dec 1;8:1880. doi: 10.1038/s41467-017-02016-y (PMC5709366; doi:10.1038/s41467-017-02016-y)
Supplement: Supplementary file 1 — Supplementary Information [file 41467_2017_2016_MOESM1_ESM.pdf]

Supplementary Note 1. Simulation of H_{c2} and magnetoresistivity using solutions of Eilenberger equations

We used Eilenberger equations in the clean limit approximation to calculate all necessary properties of the superconducting condensate. The Eilenberger equations [3] and gap equation are

$$\left(\varepsilon_n + \frac{1}{2}\hbar \mathbf{v_F} \cdot \mathbf{\Pi}\right) f(\varepsilon_n, \mathbf{r}, \mathbf{k}) = \Delta(\mathbf{r}, \mathbf{k}) g(\varepsilon_n, \mathbf{r}, \mathbf{k}) \quad (1)$$

$$\left(\varepsilon_n + \frac{1}{2}\hbar \mathbf{v_F} \cdot \mathbf{\Pi}^*\right) f^+(\varepsilon_n, \mathbf{r}, \mathbf{k}) = \Delta^*(\mathbf{r}, \mathbf{k}) g(\varepsilon_n, \mathbf{r}, \mathbf{k}) \quad (2)$$

$$\Delta(\mathbf{r}, \mathbf{k}) = -2\pi T \sum_{n=0}^{N_c(T)} \int_{\mathrm{FS}} V(\mathbf{k}, \mathbf{k}') f(\varepsilon_n, \mathbf{r}, \mathbf{k}') \frac{1}{(2\pi)^3 |\mathbf{v_F}|} \mathrm{d}S_{\mathbf{k}'}, \quad (3)$$

supplemented by the equation

$$g^2(\varepsilon_n, \mathbf{r}, \mathbf{k}) + f(\varepsilon_n, \mathbf{r}, \mathbf{k}) f^+(\varepsilon_n, \mathbf{r}, \mathbf{k}) = 1 \ , \quad (4)$$

where $N_{\mathrm{c}}(T) = \Omega_{\mathrm{BCS}}/2\pi T$ is the a cutoff in the number of states correspondent to the cutoff energy Ω_{BCS}, Fermi Surface (FS) stands for Fermi surface, $\mathrm{d}S_{\mathbf{k}'}$ is an infinitesimal element of the Fermi surface, $\varepsilon_n = (2n+1)\pi T$ are the Matsubara frequencies, T is the temperature, $\hbar$ is the reduced Planck constant, $\mathbf{\Pi} = \left(\mathbf{\nabla} - i\frac{2\pi}{\Phi_0}\mathbf{A}\right)$, Φ_0 is the magnetic flux quantum, $\mathbf{A}$ is the magnetic vector potential, $f(\varepsilon_n, \mathbf{r}, \mathbf{k})$, $f^+(\varepsilon_n, \mathbf{r}, \mathbf{k})$, and $g(\varepsilon_n, \mathbf{r}, \mathbf{k})$ are the anomalous and the conventional quasiclassical Green functions, respectively. $\Delta(\mathbf{r}, \mathbf{k})$ is the superconducting gap function that depends on the spatial position $\mathbf{r}$ and on the internal momentum $\mathbf{k}$ of the Cooper pairs, $V(\mathbf{k}, \mathbf{k}')$ is the pairing potential, and $\mathbf{v_F}$ is the Fermi velocity. H_{c2} can be found by solving the linearized version of these equations (by making $f(\varepsilon_n, \mathbf{r}, \mathbf{k}) \approx f^{(1)}(\varepsilon_n, \mathbf{r}, \mathbf{k})$ and $g(\varepsilon_n, \mathbf{r}, \mathbf{k}) \approx 1$), i.e.

$$\left(\varepsilon_n + \frac{1}{2}\hbar \mathbf{v_F} \cdot \vec{\Pi}\right) f^{(1)}(\varepsilon_n, \mathbf{r}, \mathbf{k}) = \Delta(\mathbf{r}, \mathbf{k}) \quad (5)$$

$$\Delta(\mathbf{r}, \mathbf{k}) = -2\pi T \sum_{n=0}^{N_c} \int_{FS} V(\mathbf{k}, \mathbf{k}') f^{(1)}(\varepsilon_n, \mathbf{r}, \mathbf{k}') \frac{1}{(2\pi)^3 |\mathbf{v_F}|} \mathrm{d}S_{\mathbf{k}'}. \quad (6)$$

We note that near H_{c2} the induced currents are very small and can be neglected, thus, the magnetic field in the superconductor is equal to the applied magnetic field ($\mathbf{B_a}$), i.e.

$$\mathbf{\nabla} \times \mathbf{A} = \mathbf{B_a}. \quad (7)$$

Supplementary Note 2. Parameterization of the pairing potential

The parameterization of the pairing potential was constructed using a procedure similar to the ones found in Ref. [4] and Ref. [5]. We decomposed the pairing potential, $V(\mathbf{k},\mathbf{k}')$, superconducting condensate function, $\Delta(\mathbf{r},\mathbf{k})$ and anomalous Green functions, $f(\varepsilon_n,\mathbf{r},\mathbf{k})$ in a set of basis functions dependent on $\mathbf{k}$ that diagonalize the pairing potential, i.e.

$$V(\mathbf{k},\mathbf{k}') = \sum_{i=1}^{N_q} \bar{g}_i \bar{\phi}_i(\mathbf{k}) \bar{\phi}_i(\mathbf{k}'), \quad (8)$$

where N_q is the number of components of the superconducting condensate. We note these functions are orthogonal and normalized,

$$\langle \bar{\phi}_i(\mathbf{k}) \bar{\phi}_j(\mathbf{k}) \rangle = \delta_{ij}, \quad (9)$$

using the norm

$$\langle \phi_i(\mathbf{k}) \phi_j(\mathbf{k}) \rangle = \int_{FS} \bar{\phi}_i(\mathbf{k}) \bar{\phi}_j(\mathbf{k}) \frac{1}{(2\pi)^3 N |\mathbf{v}_\mathbf{F}|} \mathrm{d}S_{\mathbf{k}'}. \quad (10)$$

Near T_c we can approximate the pairing potential, $V(\mathbf{k},\mathbf{k}')$, by the products of the form $\bar{g}_j \bar{\phi}_j(\mathbf{k}) \bar{\phi}_j(\mathbf{k}')$ where $\bar{g}_j$ correspond to critical temperatures, T_{cj} , in the neighborhood of the material's critical temperature T_c. Taking into account experimental evidences found in literature, we assumed a pairing potential with two dominant components, $s_\pm$-wave and $d_{x^2-y^2}$-wave, for which we have assigned the functions, $\bar{\phi}_1(\mathbf{k})$ and $\bar{\phi}_2(\mathbf{k})$, respectively. In this case, the pairing potential can be approximated by

$$V(\mathbf{k},\mathbf{k}') = \sum_{i=1}^{2} \bar{g}_i \bar{\phi}_i(\mathbf{k}) \bar{\phi}_i(\mathbf{k}'), \quad (11)$$

and the expansion of the superconducting condensate function by

$$\Delta(\mathbf{r},\mathbf{k}) = \sum_{j=1}^{2} \bar{\phi}_j(\mathbf{k}) \bar{\Delta}_j(\mathbf{r}), \quad (12)$$

where $\bar{\Delta}_1(\mathbf{r})$ and $\bar{\Delta}_2(\mathbf{r})$ are the spatial components of the gap function associated with the symmetries $s_\pm$- and $d_{x^2-y^2}$-wave, respectively. Additionally, we inserted couplings between the different components that might arise from small anisotropic distribution of the doping atoms or small strains in the sample. In this case the pairing potential transforms into

$$V(\mathbf{k},\mathbf{k}') = \sum_{i=1}^{2} \bar{g}_i \bar{\phi}_i(\mathbf{k}) \bar{\phi}_i(\mathbf{k}') + \bar{g}_{12} \bar{\phi}_1(\mathbf{k}) \bar{\phi}_2(\mathbf{k}') + \bar{g}_{12} \bar{\phi}_2(\mathbf{k}) \bar{\phi}_1(\mathbf{k}'), \quad (13)$$

where $\bar{g}_{12}$ is the scattering rate between the $s_\pm$- and $d_{x^2-y^2}$-wave components of the order parameter. $V(\mathbf{k},\mathbf{k}')$ is no longer in the form displayed in Supplementary Eq. (8) due to the scattering between the components. However, we can transform it back into that

form by taking linear combinations ϕ_1 and ϕ_2 of the previous $\bar{\phi}_1$ and $\bar{\phi}_2$ functions. The paring potential becomes

$$V(\mathbf{k},\mathbf{k}') = \sum_{i=1}^{2} g_i\phi_i(\mathbf{k})\phi_i(\mathbf{k}'), \tag{14}$$

and the superconducting condensate function maintain the same form in respect to the new variables, i.e $\Delta(\mathbf{r},\mathbf{k}) = \sum_{j=1}^{2} \phi_j(\mathbf{k})\Delta_j(\mathbf{r})$, where the $\Delta_1(\mathbf{r})$ and $\Delta_2(\mathbf{r})$ are linear combinations of $\overline{\Delta}_1(\mathbf{r})$ and $\overline{\Delta}_2(\mathbf{r})$ and g_1 and g_2 are constants associated with new ϕ_1 and ϕ_2 functions, respectively. Inserting these approximations into the linearized Eilenberger equations, we obtain

$$\left(\varepsilon_n + \frac{1}{2}\hbar\mathbf{v}_\mathbf{F}\cdot\mathbf{\Pi}\right) f^{(1)}(\varepsilon_n,\mathbf{r},\mathbf{k}) = \sum_{j=1}^{2} \phi_j(\mathbf{k})\Delta_j(\mathbf{r}) \tag{15}$$

$$\Delta_l(\mathbf{r}) = 2\pi g_l T \sum_{n=0}^{N_c(T)} \int_{FS} \phi_l(\mathbf{k}') f^{(1)}(\varepsilon_n,\mathbf{r},\mathbf{k}') \frac{1}{(2\pi)^3|\mathbf{v}_\mathbf{F}|} \mathrm{d}S_{\mathbf{k}'}. \tag{16}$$

To obtain $\bar{\phi}_j(\mathbf{k})$ for $Ba_{1-x}K_xFe_2As_2$, we expanded it in harmonic functions centered in each pocket of the Fermi surface, in a similar procedure to the one described in Ref. [5]. The selected harmonic functions are compliant with the symmetry of pocket's geometry and of the corresponding $\bar{\phi}_j(\mathbf{k})$, i.e.

$$\bar{\phi}_{1,h1}(\hat{\theta}) = \bar{\phi}_{1,h2}(\hat{\theta}) = \bar{\phi}_{1,h3}(\hat{\theta}) = 1 \tag{17}$$

$$\bar{\phi}_{2,h1}(\hat{\theta}) = \bar{\phi}_{2,h2}(\hat{\theta}) = \bar{\phi}_{2,h3}(\hat{\theta}) = \cos(2\hat{\theta}), \tag{18}$$

and

$$\bar{\phi}_{1,e1}(\hat{\theta}) = a_{1,1} + a_{1,2}\cos(2\hat{\theta}) \tag{19}$$

$$\bar{\phi}_{1,e2}(\hat{\theta}) = a_{1,1} - a_{1,2}\cos(2\hat{\theta}) \tag{20}$$

$$\bar{\phi}_{2,e1}(\hat{\theta}) = a_{2,1}\cos(2\hat{\theta}) + a_{2,2} \tag{21}$$

$$\bar{\phi}_{2,e2}(\hat{\theta}) = a_{2,1}\cos(2\hat{\theta}) - a_{2,2}, \tag{22}$$

where $\hat{\theta}$ is an angle defined according to a fixed lattice axis and the center of the respective pocket, $h1$, $h2$ and $h3$ correspond to the inner, the outer central hole pockets and corner hole pockets and $e1$ and $e2$ correspond to the electron pockets on the sides of the Fermi surface, all of which can be identified in Supplementary Figure 18 for the case of $Ba_{0.5}K_{0.5}Fe_2As_2$.

We assume that $\bar{\phi}_j(\mathbf{k})$ is evenly distributed over the Fermi surfaces of the central holes and electron pockets, which is a good approximation since the interband scattering is stronger than the intra-band scattering between the central hole pockets and the

electron pockets. However, we made a broader assumption, which was to consider that $\bar{\phi}_j(\mathbf{k})$ is evenly distributed over all pockets of the Fermi surface.

For convenience is better to express $\bar{\lambda}_1 = -\bar{g}_1 N$, $\bar{\lambda}_2 = -\bar{g}_2 N$ and $\bar{\gamma}_{12} = -\bar{g}_{12} N$ as functions of T_{c1} and T_{c2} and $\lambda_1 = -g_1 N$ and $\lambda_2 = -g_2 N$ as functions of T'_{c1} and T'_{c2}.

Usual BCS equation (which can be obtained from Supplementary Eq. (3)) for a homogeneous single component superconductor (this means that $\Delta = const.$ and $V(\mathbf{k}, \mathbf{k}') = g$) is

$$\Delta = 2\pi T \sum_{n=0}^{N_c(T_c)} \int_{FS} \frac{\lambda}{(2\pi)^3 N |\mathbf{v}_\mathrm{F}|} \frac{\Delta}{\sqrt{\Delta^2 + \varepsilon_n^2}} \mathrm{d}S_{\mathbf{k}'}. \quad (23)$$

By considering that $T \to T_\mathrm{c}$ then $\Delta \to 0$ and as usual we obtain

$$\frac{1}{\lambda} = 2\pi T \sum_{n=0}^{N_\mathrm{c}(T_c)} \frac{1}{\varepsilon_n}. \quad (24)$$

Using it, the following sum can separated into

$$2\pi T \sum_{n=0}^{N_\mathrm{c}(T)} \frac{1}{\varepsilon_n} = 2\pi T \sum_{n=0}^{N_\mathrm{c}(T_c)} \frac{1}{\varepsilon_n} + 2\pi T \sum_{n=N_\mathrm{c}(T_\mathrm{c})}^{N_c(T)} \frac{1}{\varepsilon_n}$$

$$= \frac{1}{\lambda} + \sum_{n=N_c(T_c)}^{N_c(T)} \frac{1}{n+1/2}. \quad (25)$$

This sum can also be approximated by

$$\sum_{n=0}^{N_\mathrm{c}(T)} \frac{1}{n+1/2} \approx \ln(N_0(T)) + 2\ln(2) + \mathrm{C} \quad (26)$$

in the case of large $N_\mathrm{c}(T)$, where $N_\mathrm{c}(T) = \Omega_\mathrm{BCS}/2\pi T$ and $\mathrm{C} = 0.5772\ldots$ (Euler's constant). Using the previous expression we obtain

$$\sum_{n=N_\mathrm{c}(T_\mathrm{c})}^{N_\mathrm{c}(T)} \frac{1}{n+1/2} \approx \ln\left(\frac{T_\mathrm{c}}{T}\right). \quad (27)$$

and thus

$$2\pi T \sum_{n=0}^{N_\mathrm{c}(T)} \frac{1}{\varepsilon_n} \approx \frac{1}{\lambda} + \ln\left(\frac{T_\mathrm{c}}{T}\right), \quad (28)$$

from which we get

$$\frac{\Delta(\mathbf{r})}{\lambda} - 2\pi T \sum_{n=0}^{N_\mathrm{c}(T)} \frac{\Delta(\mathbf{r})}{\varepsilon_n} = \frac{\Delta(\mathbf{r})}{\lambda} - \frac{\Delta(\mathbf{r})}{\lambda} - \ln\left(\frac{T_c}{T}\right)\Delta(\mathbf{r}) \approx -\ln\left(\frac{T_c}{T}\right)\Delta(\mathbf{r}). \quad (29)$$

Supplementary Eq. (16),

$$\Delta_l(\mathbf{r}) = 2\pi\lambda_l T \sum_{n=0}^{N_c(T)} \int_{FS} \phi_l(\mathbf{k}') f^{(1)}(\varepsilon_n, \mathbf{r}, \mathbf{k}') \frac{1}{(2\pi)^3 |\mathbf{v}_\mathrm{F}| N} \mathrm{d}S_{\mathbf{k}'}, \quad (30)$$

can be approximated to

$$\Delta_l(\mathbf{r}) = 2\pi\lambda_l T \left(\sum_{n=0}^{N_\mathrm{c}(T)} \frac{\Delta_l(\mathbf{r})}{\varepsilon_n} - \sum_{n=0}^{\infty} \frac{\Delta_l(\mathbf{r})}{\varepsilon_n} + \right.$$

$$\sum_{n=0}^{\infty} \int_{FS} \phi_l(\mathbf{k}') f^{(1)}(\varepsilon_n, \mathbf{r}, \mathbf{k}') \frac{1}{(2\pi)^3 |\mathbf{v_F}| N} \mathrm{d}S_{\mathbf{k}'} \Big), \quad (31)$$

and combining it with Supplementary Eq. (29) we obtain

$$\Delta_l(\mathbf{r}) \ln\left(\frac{T'_{cl}}{T}\right) = 2\pi T \left(\sum_{n=0}^{\infty} \int_{FS} \phi_l(\mathbf{k}') f^{(1)}(\varepsilon_n, \mathbf{r}, \mathbf{k}') \frac{1}{(2\pi)^3 |\mathbf{v_F}| N} \mathrm{d}S_{\mathbf{k}'} - \sum_{n=0}^{\infty} \frac{\Delta_l(\mathbf{r})}{\varepsilon_n} \right). \quad (32)$$

For simplicity we took the same cutoff energy, Ω_{BCS}, for all components.

Supplementary Note 3. Expansion into Landau levels and H_{c2} of two-component superconductors

We made a perturbation expansion of the first equation with respect to the operator $\mathbf{v_F} \cdot \mathbf{\Pi}$. The v-th order of perturbation of the anomalous Green function is,

$$f_v^{(1)}(\varepsilon_n, \mathbf{r}, \mathbf{k}) = \delta_{v0} \frac{\sum_{j=1}^{\infty} \phi_j(\mathbf{k}) \Delta_j(\mathbf{r})}{\varepsilon_n} - \frac{1}{2\varepsilon_n} \hbar \mathbf{v_F} \cdot \mathbf{\Pi} f_{v-1}^{(1)}(\varepsilon_n, \mathbf{r}, \mathbf{k}). \quad (33)$$

Inserting this expansion from the zero-th to the fourth order into the gap equation (Supplementary Eq. (32)), it becomes

$$\Delta_1 \ln\left(\frac{T'_{c1}}{T}\right) = -2\pi T \sum_{n=0}^{\infty} \Bigg[\left\langle \phi_1^2 \left(\frac{1}{2\varepsilon_n} \hbar \mathbf{v_F} \cdot \mathbf{\Pi}\right)^2 \Delta_1 \right\rangle + \left\langle \phi_1 \phi_2 \left(\frac{1}{2\varepsilon_n} \hbar \mathbf{v_F} \cdot \mathbf{\Pi}\right)^2 \Delta_2 \right\rangle + \\ + \left\langle \phi_1 \phi_2 \left(\frac{1}{2\varepsilon_n} \hbar \mathbf{v_F} \cdot \mathbf{\Pi}\right)^3 \Delta_2 \right\rangle + \left\langle \phi_1^2 \left(\frac{1}{2\varepsilon_n} \hbar \mathbf{v_F} \cdot \mathbf{\Pi}\right)^4 \Delta_1 \right\rangle + \left\langle \phi_1 \phi_2 \left(\frac{1}{2\varepsilon_n} \hbar \mathbf{v_F} \cdot \mathbf{\Pi}\right)^4 \Delta_2 \right\rangle \Bigg], \quad (34)$$

$$\Delta_2 \ln\left(\frac{T'_{c2}}{T}\right) = -2\pi T \sum_{n=0}^{\infty} \Bigg[\left\langle \phi_2^2 \left(\frac{1}{2\varepsilon_n} \hbar \mathbf{v_F} \cdot \mathbf{\Pi}\right)^2 \Delta_2 \right\rangle + \left\langle \phi_1 \phi_2 \left(\frac{1}{2\varepsilon_n} \hbar \mathbf{v_F} \cdot \mathbf{\Pi}\right)^2 \Delta_1 \right\rangle + \\ + \left\langle \phi_1 \phi_2 \left(\frac{1}{2\varepsilon_n} \hbar \mathbf{v_F} \cdot \mathbf{\Pi}\right)^3 \Delta_1 \right\rangle + \left\langle \phi_2^2 \left(\frac{1}{2\varepsilon_n} \hbar \mathbf{v_F} \cdot \mathbf{\Pi}\right)^4 \Delta_2 \right\rangle + \left\langle \phi_1 \phi_2 \left(\frac{1}{2\varepsilon_n} \hbar \mathbf{v_F} \cdot \mathbf{\Pi}\right)^4 \Delta_1 \right\rangle \Bigg], \quad (35)$$

Afterwards, we expressed the operator $\mathbf{v_F} \cdot \mathbf{\Pi}$ in terms of ladder operators associated with the Landau levels of Δ_1 and Δ_2, i.e.

$$\mathbf{v_F} \cdot \mathbf{\Pi} \Delta_l(\mathbf{r}) = \frac{1}{\sqrt{2} l_c} \left(\mathbf{v}_{\mathbf{F},\mathbf{l}}^* a_l - \mathbf{v}_{\mathbf{F},\mathbf{l}} a_l^\dagger \right) \Delta_l(\mathbf{r}), \quad (36)$$

where $l_c = \sqrt{\Phi_0 / 2\pi B}$,

$$\mathbf{v}_{\mathbf{F},\mathbf{l}} = c_{2,l} v_{Fx} + i c_{1,l} v_{Fy}, \quad (37)$$

and a_l and $a_l^\dagger$ lower and raise one Landau level in the l -th component, i.e.

$$a_l |n\rangle_l = \sqrt{n} |n-1\rangle_l,$$
$$a_l^\dagger |n\rangle_l = \sqrt{n+1} |n+1\rangle_l.$$

These ladder operators are related to $\mathbf{\Pi}$ by

$$\begin{pmatrix} a_1 \\ a_1^{\dagger} \end{pmatrix} = \frac{l_c}{\sqrt{2}} \begin{pmatrix} c_{1,1} & i c_{2,1} \\ -c_{1,1}^{*} & i c_{2,1}^{*} \end{pmatrix} \begin{pmatrix} \Pi_x \\ \Pi_y \end{pmatrix}, \tag{38}$$

$$\begin{pmatrix} a_2 \\ a_2^{\dagger} \end{pmatrix} = \frac{l_c}{\sqrt{2}} \begin{pmatrix} c_{1,2} & i c_{2,2} \\ -c_{1,2}^{*} & i c_{2,2}^{*} \end{pmatrix} \begin{pmatrix} \Pi_x \\ \Pi_y \end{pmatrix}. \tag{39}$$

The coordinate system (x, y, z) is chosen such that $\mathbf{z}$ is along $\mathbf{H}$. A coordinate system, independent of $\mathbf{H}$, can be chosen along a, b and c-axes, where the coordinates along these directions are (X, Y, Z). In this new coordinate system, the previous Fermi velocity components can be written as

$$v_{Fx} = v_{FX}\cos(\varphi)\cos(\theta) + v_{FY}\cos(\varphi)\sin(\theta) - v_{FZ}\sin(\varphi) \tag{40}$$

$$v_{Fy} = -v_{FX}\sin(\theta) + v_{FY}\cos(\theta), \tag{41}$$

where θ and ϕ are the angles such that $\mathbf{H} = |\mathbf{H}|(\sin(\varphi)\cos(\theta), \sin(\varphi)\sin(\theta), \cos(\varphi))$.

The previous system of equations expanded in the Landau levels becomes

$$\begin{aligned} -w_{(0,0),1}\Delta_1 = {} & B[-w_{(2,0),1}(\phi_1^2)A_{(1,1),1} + w_{(2,2),1}(\phi_1^2)A_{(1,0),1}^{*} + w_{(2,2),1}^{*}(\phi_1^2)A_{(1,0),1}]\Delta_1 + \\ & B[-w_{(2,0),2}(\phi_1\phi_2)A_{(1,1),2} + w_{(2,2),2}(\phi_1\phi_2)A_{(1,0),2}^{*} + w_{(2,2),2}^{*}(\phi_1\phi_2)A_{(1,0),2}]\Delta_2 + \\ & B^{3/2}[-w_{3,1}(\phi_1\phi_2)A_{(2,1,1),l} + w_{3,2}(\phi_1\phi_2)A_{(1,2,1),l} + w_{3,3}(\phi_1\phi_2)A_{(1,2,2),l} - \\ & w_{3,4}(\phi_1\phi_2)A_{(0,3),l} + w_{3,5}(\phi_1\phi_2)A_{(3,0),l} - w_{3,6}(\phi_1\phi_2)A_{(2,1,2),l}]\Delta_2 + \\ & B^2[w_{(4,0),1}(\phi_1^2)A_{(2,2),1} - w_{(4,2),1}(\phi_1^2)A_{(3,1),1}^{*} - w_{(4,2),1}^{*}(\phi_1^2)A_{(3,1),1} + \\ & w_{(4,4),1}(\phi_1^2)A_{(4,0),1}^{*} + w_{(4,4),1}^{*}(\phi_1^2)A_{(4,0),1}]\Delta_1 + B^2[w_{(4,0),2}(\phi_1\phi_2)A_{(2,2),2} - \\ & w_{(4,2),2}(\phi_1\phi_2)A_{(3,1),2}^{*} - w_{(4,2),2}^{*}(\phi_1\phi_2)A_{(3,1),2} + w_{(4,4),2}(\phi_1\phi_2)A_{(4,0),2}^{*} + \\ & w_{(4,4),2}^{*}(\phi_1\phi_2)A_{(4,0),2}]\Delta_2 \end{aligned} \tag{42}$$

$$\begin{aligned} -w_{(0,0),2}\Delta_2 = {} & B[-w_{(2,0),2}(\phi_2^2)A_{(1,1),2} + w_{(2,2),2}(\phi_2^2)A_{(1,0),2}^{*} + w_{(2,2),2}^{*}(\phi_2^2)A_{(1,0),2}]\Delta_2 + \\ & B[-w_{(2,0),1}(\phi_1\phi_2)A_{(1,1),1} + w_{(2,2),1}(\phi_1\phi_2)A_{(1,0),1}^{*} + w_{(2,2),1}^{*}(\phi_1\phi_2)A_{(1,0),1}]\Delta_1 + \\ & B^{3/2}[-w_{3,1}(\phi_1\phi_2)A_{(2,1,1),l} + w_{3,2}(\phi_1\phi_2)A_{(1,2,1),l} + w_{3,3}(\phi_1\phi_2)A_{(1,2,2),l}S - \\ & w_{3,4}(\phi_1\phi_2)A_{(0,3),l} + w_{3,5}(\phi_1\phi_2)A_{(3,0),l} - w_{3,6}(\phi_1\phi_2)A_{(2,1,2),l}]\Delta_1 + \\ & B^2[w_{(4,0),2}(\phi_2^2)A_{(2,2),2} - w_{(4,2),2}(\phi_2^2)A_{(3,1),2}^{*} - w_{(4,2),2}^{*}(\phi_2^2)A_{(3,1),2} + \\ & w_{(4,4),2}(\phi_2^2)A_{(4,0),2}^{*} + w_{(4,4),2}^{*}(\phi_2^2)A_{(4,0),2}]\Delta_2 + \\ & B^2[w_{(4,0),1}(\phi_1\phi_2)A_{(2,2),1} - w_{(4,2),1}(\phi_1\phi_2)A_{(3,1),1}^{*} - w_{(4,2),1}^{*}(\phi_1\phi_2)A_{(3,1),1} + \end{aligned}$$

$$w_{(4,4),1}(\phi_1\phi_2)A^*_{(4,0),1} + w^*_{(4,4),1}(\phi_1\phi_2)A_{(4,0),1}]\Delta_1. \qquad (43)$$

where

$$A_{(2,1,1),l} = (a_l a_l^\dagger + a_l^\dagger a_l)a_l + a_l(a_l a_l^\dagger + a_l^\dagger a_l)$$

$$A_{(1,2,1),l} = (a_l a_l^\dagger + a_l^\dagger a_l)a_l^\dagger + a_l^\dagger(a_l a_l^\dagger + a_l^\dagger a_l)$$

$$A_{(1,2,2),l} = (a_l^\dagger)^2 a_l + a_l(a_l^\dagger)^2$$

$$A_{(0,3),l} = (a_l^\dagger)^2 a_l^\dagger + a_l^\dagger(a_l^\dagger)^2]$$

$$A_{(3,0),l} = a_l^2 a_l + a_l a_l^2$$

$$A_{(2,1,2),l} = a_l^2 a_l^\dagger + a_l^\dagger a_l^2$$

$$A_{(1,3),l} = a_l a_l^\dagger a_l^\dagger a_l^\dagger + a_l^\dagger a_l^\dagger a_l^\dagger a_l + a_l^\dagger a_l^\dagger a_l a_l^\dagger + a_l^\dagger a_l a_l^\dagger a_l$$

$$A_{(3,1),l} = a_l a_l^\dagger a_l a_l + a_l^\dagger a_l a_l a_l + a_l a_l a_l a_l^\dagger + a_l a_l a_l^\dagger a_l$$

$$A_{(2,2),l} = a_l a_l^\dagger a_l^\dagger a_l + a_l^\dagger a_l a_l a_l^\dagger + a_l a_l^\dagger a_l a_l^\dagger + a_l^\dagger a_l a_l^\dagger a_l$$

$$+a_l a_l a_l^\dagger a_l^\dagger + a_l^\dagger a_l^\dagger a_l a_l$$

$$A_{(0,4),l} = a_l^\dagger a_l^\dagger a_l^\dagger a_l^\dagger$$

$$A_{(4,0),l} = a_l a_l a_l a_l$$

$$A_{(1,1),l} = a_l a_l^\dagger + a_l^\dagger a_l$$

$$A_{(1,0),l} = a_l^2$$

$$A_{(0,1),l} = (a_l^\dagger)^2,$$

and

$$w_{(2,2),l}(f(\mathbf{k})) = \sum_{n=0}^{\infty} \frac{(2\pi)^2 T\hbar^2}{2^3\varepsilon_n^3\Phi_0}\langle f(\mathbf{k})(\bar{v}_{F,l})^2\rangle$$

$$w^*_{(2,2),l}(f(\mathbf{k})) = \sum_{n=0}^{\infty} \frac{(2\pi)^2 T\hbar^2}{2^3\varepsilon_n^3\Phi_0}\langle f(\mathbf{k})(\bar{v}^*_{F,l})^2\rangle$$

$$w_{(2,0),l}(f(\mathbf{k})) = \sum_{n=0}^{\infty} \frac{(2\pi)^2 T\hbar^2}{2^3\varepsilon_n^3\Phi_0}\langle f(\mathbf{k})|\bar{v}_{F,l}|^2\rangle$$

$$w_{(3,1),l}(f(\mathbf{k})) = \sum_{n=0}^{\infty} \frac{(2\pi)^{5/2} T\hbar^3}{2^{9/2}\varepsilon_n^4\Phi_0^{3/2}}\langle f(\mathbf{k})|\bar{v}_{F,l}|^2\bar{v}^*_{F,l}\rangle$$

$$w_{(3,2),l}(f(\mathbf{k})) = \sum_{n=0}^{\infty} \frac{(2\pi)^{5/2} T\hbar^3}{2^{9/2}\varepsilon_n^4 \Phi_0^{3/2}} \langle f(\mathbf{k}) |\bar{v}_{F,l}|^2 \bar{v}_{F,l} \rangle$$

$$w_{(3,3),l}(f(\mathbf{k})) = \sum_{n=0}^{\infty} \frac{(2\pi)^{5/2} T\hbar^3}{2^{9/2}\varepsilon_n^4 \Phi_0^{3/2}} \langle f(\mathbf{k}) (\bar{v}_{F,l})^2 \bar{v}_{F,l}^* \rangle$$

$$w_{(3,4),l}(f(\mathbf{k})) = \sum_{n=0}^{\infty} \frac{(2\pi)^{5/2} T\hbar^3}{2^{9/2}\varepsilon_n^4 \Phi_0^{3/2}} \langle f(\mathbf{k}) (\bar{v}_{F,l})^2 \bar{v}_{F,l} \rangle$$

$$w_{(3,5),l}(f(\mathbf{k})) = \sum_{n=0}^{\infty} \frac{(2\pi)^{5/2} T\hbar^3}{2^{9/2}\varepsilon_n^4 \Phi_0^{3/2}} \langle f(\mathbf{k}) (\bar{v}_{F,l}^*)^2 \bar{v}_{F,l}^* \rangle$$

$$w_{(3,6),l}(f(\mathbf{k})) = \sum_{n=0}^{\infty} \frac{(2\pi)^{5/2} T\hbar^3}{2^{9/2}\varepsilon_n^4 \Phi_0^{3/2}} \langle f(\mathbf{k}) (\bar{v}_{F,l}^*)^2 \bar{v}_{F,l} \rangle$$

$$w_{(4,0),l}(f(\mathbf{k})) = \sum_{n=0}^{\infty} \frac{(2\pi)^3 T\hbar^4}{2^6 \varepsilon_n^5 \Phi_0^2} \langle f(\mathbf{k}) |\bar{v}_{F,l}|^4 \rangle$$

$$w_{(4,2),l}(f(\mathbf{k})) = \sum_{n=0}^{\infty} \frac{(2\pi)^3 T\hbar^4}{2^6 \varepsilon_n^5 \Phi_0^2} \langle f(\mathbf{k}) |\bar{v}_{F,l}|^2 (\bar{v}_{F,l})^2 \rangle$$

$$w_{(4,2),l}^*(f(\mathbf{k})) = \sum_{n=0}^{\infty} \frac{(2\pi)^3 T\hbar^4}{2^6 \varepsilon_n^5 \Phi_0^2} \langle f(\mathbf{k}) |\bar{v}_{F+}|^2 (\bar{v}_{F,l}^*)^2 \rangle$$

$$w_{(4,4),l}(f(\mathbf{k})) = \sum_{n=0}^{\infty} \frac{(2\pi)^3 T\hbar^4}{2^6 \varepsilon_n^5 \Phi_0^2} \langle f(\mathbf{k}) (\bar{v}_{F,l})^4 \rangle$$

$$w_{(4,4),l}^*(f(\mathbf{k})) = \sum_{n=0}^{\infty} \frac{(2\pi)^3 T\hbar^4}{2^6 \varepsilon_n^5 \Phi_0^2} \langle f(\mathbf{k}) (\bar{v}_{F,l}^*)^4 \rangle$$

$$w_{(0,0),l} = \ln\left(\frac{T_{cl}'}{T}\right).$$

The values of variables $c_{1,1}$, $c_{2,1}$, $c_{1,2}$ and $c_{2,2}$ are arbitrary and we have chosen these such that $w_{2,2}(\phi_1^2) = 0$ and $w_{2,2}(\phi_2^2) = 0$. Projecting the first and second equation into the lowest Landau levels Δ_1^0 and Δ_2^0, respectively, we simplified the previous equations to

$$\begin{aligned} 0 = {} & w_{(0,0),1} - B_{c2}[w_{(2,0),1}(\phi_1^2)] - B_{c2}[w_{(2,0),2}(\phi_1\phi_2)_1\langle 0|0\rangle_2] \\ & +3B_{c2}^2[w_{(4,0),1}(\phi_1^2)] + 3B_{c2}^2[w_{(4,0),2}(\phi_1\phi_2)_1\langle 0|0\rangle_2] \end{aligned} \quad (44)$$

$$\begin{aligned} 0 = {} & w_{(0,0),2} - B_{c2}[w_{(2,0),2}(\phi_2^2)] - B_{c2}[w_{(2,0),1}(\phi_1\phi_2)_2\langle 0|0\rangle_1] \\ & +3B_{c2}^2[w_{(4,0),2}(\phi_2^2)] + 3B_{c2}^2[w_{(4,0),1}(\phi_1\phi_2)_2\langle 0|0\rangle_1], \end{aligned} \quad (45)$$

where $|0\rangle_1$ and $|0\rangle_2$ are the Dirac ket's directly corresponding to the Landau levels of Δ_1^0 and Δ_2^0, respectively, and B was replaced by B_{c2} since, by definition, it is the second critical field corresponding to a given T .

Supplementary Note 4. H_{c2} of three-component superconductors

The experimental curves display other features rather that the strong anisotropy close to Fe-Fe bond direction that cannot be explained within the two-component model constructed previously. To refined our model we add a third component $\bar{\phi}_3(\mathbf{k})$ with a symmetry different from the one on $\bar{\phi}_1(\mathbf{k})$ and $\bar{\phi}_2(\mathbf{k})$ into the pairing potential which becomes

$$V(\mathbf{k},\mathbf{k}') = \sum_{i=1}^{3} \bar{g}_i \bar{\phi}_i(\mathbf{k})\bar{\phi}_i(\mathbf{k}') + \sum_{i=1}^{3} \sum_{j=1}^{3} \bar{g}_{ij} \bar{\phi}_i(\mathbf{k})\bar{\phi}_j(\mathbf{k}') \quad (46)$$

and can be transformed into the form

$$V(\mathbf{k},\mathbf{k}') = \sum_{i=1}^{3} g_i \phi_i(\mathbf{k})\phi_i(\mathbf{k}'), \quad (47)$$

as in the case of two-components. The symbols $\bar{g}_{ij}$ are the scattering constants between the i component and the j component, also $\bar{g}_{ij} = \bar{g}_{ji}$ and $\bar{\phi}_1(\mathbf{k}), \overline{\phi}_2(\mathbf{k})$ are functions given by Supplementary Eqs. (17)-(22). In the case of third component is of g-wave symmetry, $\bar{\phi}_3(\mathbf{k})$ is given by

$$\bar{\phi}_{3,h1}(\hat{\theta}) = \sin(4\hat{\theta}) \quad (48)$$

$$\bar{\phi}_{3,e1}(\hat{\theta}) = a_{3,1}\sin(4\hat{\theta}) + a_{3,2}\sin(2\hat{\theta}) \quad (49)$$

$$\bar{\phi}_{3,e1}(\hat{\theta}) = a_{3,1}\sin(4\hat{\theta}) + a_{3,2}\sin(2\hat{\theta}) \quad (50)$$

and, in the case of d_{xy}-wave symmetry, $\bar{\phi}_3(\mathbf{k})$ is given by

$$\bar{\phi}_{3,h1}(\hat{\theta}) = \sin(2\hat{\theta}) \quad (51)$$

$$\bar{\phi}_{3,e1}(\hat{\theta}) = a_{3,1}\sin(2\hat{\theta}) + a_{3,2}\sin(4\hat{\theta}) \quad (52)$$

$$\bar{\phi}_{3,e2}(\hat{\theta}) = a_{3,1}\sin(2\hat{\theta}) - a_{3,2}\sin(4\hat{\theta}). \quad (53)$$

Proceeding a similar way to the two component case presented above we can obtain a system of equations equivalent to Supplementary Eqs. (36) and Eq. (37) for the three-component case. The new equations are

$$0 = w_{(0,0),1} - B_{c2}\left[w_{(2,0),1}(\phi_1^2)\right] - B_{c2}[w_{(2,0),2}(\phi_1\phi_2)_1\langle 0|0\rangle_2] - B_{c2}[w_{(2,0),3}(\phi_1\phi_3)_1\langle 0|0\rangle_3] + 3B_{c2}^2[w_{(4,0),1}(\phi_1^2)] + 3B_{c2}^2[w_{(4,0),2}(\phi_1\phi_2)_1\langle 0|0\rangle_2] + 3B_{c2}^2[w_{(4,0),3}(\phi_1\phi_3)_1\langle 0|0\rangle_3] \quad (54)$$

$$0 = w_{(0,0),2} - B_{c2}[w_{(2,0),2}(\phi_2^2)] - B_{c2}[w_{(2,0),1}(\phi_1\phi_2)_2\langle 0|0\rangle_1] - B_{c2}[w_{(2,0),3}(\phi_3\phi_2)_2\langle 0|0\rangle_3] + 3B_{c2}^2[w_{(4,0),2}(\phi_2^2)] + 3B_{c2}^2[w_{(4,0),1}(\phi_1\phi_2)_2\langle 0|0\rangle_1] + 3B_{c2}^2[w_{(4,0),3}(\phi_3\phi_2)_2\langle 0|0\rangle_3] \quad (55)$$

$0 = w_{(0,0),3} - B_{c2}[w_{(2,0),3}(\phi_3^2)] - B_{c2}[w_{(2,0),2}(\phi_3\phi_2)_3\langle 0|0\rangle_2] -$
$B_{c2}[w_{(2,0),1}(\phi_3\phi_1)_3\langle 0|0\rangle_1] + 3B_{c2}^2[w_{(4,0),3}(\phi_3^2)] + 3B_{c2}^2[w_{(4,0),2}(\phi_3\phi_2)_3\langle 0|0\rangle_2 +$
$3B_{c2}^2[w_{(4,0),1}(\phi_3\phi_1)_3\langle 0|0\rangle_1]$, (56)

where $|0\rangle_3$ is the Dirac ket's corresponding to the lowest Landau level of the new component.

Supplementary Note 5. Resistivity model

The resistivity of high temperature superconductors in the vicinity of the normal - superconducting phase boundary, mainly originates from thermally activated vortex creep motion in the offset of the transition between normal to superconducting state and thermally activated flux flow in the onset of the transition region. We note that the R-T curves of our compound resemble the ones from copper-based superconductors. To simulate these experimental results, we have firstly considered Anderson-Kim's and Tinkham's models. Anderson-Kim's model models thermally activated vortex creep motion and thus can only be applied to the offset of the transition region. Tinkham's model was in the past used to fit the full normal-superconductor transition curve in copper based superconductors very successfully. We use this model here as an interpolatory model to fit to the full curve. We note that the model for magnetoresistivity as only a phenomenological character and it's details are not of critical importance for studying the qualitative features of its angular dependence. Within Anderson-Kim's and Tinkham's models, it is required to calculate the pinning barrier energy that is proportional to the free energy density of the system. Moreover, the free energy in single-band superconductors can be related to the second critical magnetic field using the Abrikosov expression [6]. In Anderson-Kim's model, the expression for resistivity is given by

$$R = \omega_0 e^{-\frac{U_0}{k_B T}}, \quad (57)$$

where ω_0 is the characteristic frequency of the flux-line vibration (from 10^5 to 10^{11}s^{-1}), and U_0 is its activation energy (or barrier's height), k_B is the Boltzmann constant, and T is the temperature. In Tinkham's model, the resistivity is given by

$$\frac{R}{R_n} = \left[I_0\left(\frac{U_0}{k_B T}\right)\right]^{-2} \quad (58)$$

where R_n is the resistivity of the normal state, I_0 is the modified Bessel function, and U_0 is the activation energy (or barrier's height). Both these models were applied by us to capture the phenomenological features of the experimental magnetoresistivity curves. However except in this subsection, we only considered Tinkham's model within the rest of the publication (including all presented plots of magnetoresistivity) since it gives a better fitting to experimental results.

In both models, U_0 can be expressed in terms of the free energy and characteristic lengths, i.e., $U_0 \propto \Delta G \frac{\Phi_0}{B} \xi(T)$ where ΔG is the Gibbs free energy, ξ is the coherence length of the condensate, Φ_0 is the magnetic flux quantum, and B is the magnitude of the induced magnetic field. We note that $\frac{\Phi_0}{B}$ is the area of a flux line (where the magnitude of the induced magnetic field can be approximated by the magnitude of the applied magnetic field, H_a) and ξ is inserted in this expression as an average length of the jump made by the magnetic fluxes.

ΔG was calculated in phenomenological form using the Abrikosov expression,

$$\Delta G = -\frac{(B_{c2} - B)^2}{8\pi(1 + (2\kappa^2 - 1)\beta_A)} \approx -\frac{B_{c2}^2}{8\pi(1 + (2\kappa^2 - 1)\beta_A)}, \quad (59)$$

where B_{c2} is the magnetic induction associated with the second critical magnetic field H_{c2} (note that we are using the CGS unit system where $H_{c2} = B_{c2}$ if we neglect the magnetic field developed by the induced currents), B is the magnitude of the magnetic field, κ is a characteristic parameter of the material, and β_A is the Abrikosov parameter that for a triangular lattice is $\beta_A = 1.16$. This expression is valid near H_{c2}, and it is obtained by considering first perturbation in the wave function and in the magnetic field to the solution of linearized Ginzburg-Landau (GL) equation in which the magnetic field is equal to the applied magnetic field as-described in the sections above. We note that we do not observe in our compound a shift in onset value of the transition (upper part of the resistivity curves in the normal-superconducting state transition) with changes in the magnetic field magnitude, as it is expected in conventional superconductors. The same behavior is observed in other high T_c superconductors like copper-based superconductors.

To take this aspect into account, we made one last approximation in Abrikosov expression, presented in Supplementary Eq. (59), as it is done in Ref. [7].

Supplementary Note 6. Simulation results of H_{c2} and magnetoresistivity and additional details

Supplementary Figure 18 shows H_{c2} for simulated and experimental values along different directions (identified by the angle θ) of the applied magnetic field. The simulated curves were calculated for two components $s_{\pm}$-wave and $d_{x^2-y^2}$ -wave symmetry alone or with a third component with g -wave or d_{xy}-wave symmetry. Both choices for third component can explain the shifting of the maximum. Supplementary Figure 20 shows the magnetoresistivity curves of the experimental curves and the simulated curves with the same components as the H_{c2} plots. Both simulations, with g-wave and d_{xy}-wave symmetries, display a magnetoresistivity with similar characteristics to the experimental curve, i.e. the maxima of the simulated curves are close to the experimental one and the mirror symmetry along the maxima is broken as it is in experiment. The simulations were conducted as described in the main text, with additional details pointed out below.

Fermi surface and Fermi velocities where obtained using tight binding five band model [8] in the rigid band approximation using Fermi level $\mu = 0.187$ eV corresponding to $n_e = 5.5$ and $x = 0.5$ in $Ba_{1-x}K_xFe_2As_2$, and consistent with Fermi levels of Ref. [9].

To calculate H_{c2} , we solved the previous system of equations numerically, considering only the solution in which $B_{c2} \to 0$ when $T \to T'_{cm}$, where T'_{cm} is the highest critical temperature of T'_{c1}, T'_{c2}, T'_{c3}. Using this solution, we have then expanded it into a Taylor series in the applied temperature, i.e. $B_{c2} = B_1(\theta)(1 - T/T_c) + B_2(\theta)(1 - [T/T_c]^2) + \cdots$., and considered only the first two terms of the expansion. In H_{c2} plots of the Ref. [10], we can see that the terms of the form $(1 - [T/T_c]^2)$ are important, and we point out that they reflect the multiband/multicomponent nature of our compound. Calculating H_{c2} for just one s-wave component using the same equations, i.e.

$$B_{c2} = \frac{\ln\left(\frac{T_c}{T}\right)}{\sum_{n=0}^{\infty} \frac{(2\pi)^2 T_c \hbar^2}{2^3 \varepsilon_n^3 \Phi_0} \langle |\mathbf{v_F}|^2 \rangle} \quad (60)$$

$$\approx \frac{\ln\left(\frac{T_c}{T}\right)}{\sum_{n=0}^{\infty} \frac{(2\pi)^2 T_c \hbar^2}{2^3 \varepsilon_n^3 \Phi_0} \langle |\widehat{\mathbf{v_F}}|^2 \rangle} \quad (61)$$

near the limit $T \rightarrow T_c$ the critical field is mainly a linear function of the temperature, i.e. $B_{c2} \propto (1 - T/T_c)$. Second critical field is highly underestimated in our compound if only of a single component is taken under consideration, forcing us to conclude that the high H_{c2} near T_c in this compound is an effect of the multiband/multicomponent nature of the material. Despite the fact the simulated H_{c2} is much closer to the experimental value when considering three components, we still cannot obtain the correct absolute value of H_{c2}, probably due to renormalization of the effective mass of electrons or the effect of more bands. To account for this discrepancy, we introduced a renormalization factor into the Fermi velocities consistent with a renormalization of the effective mass of ≈ 2.0. We note that a small measuring error in the assessment of the superconducting critical temperature T_c will have a strong impact on estimated the renormalization of the effective mass. For example if T_c shifts to a higher temperature just by 0.1 K, the renormalization value will be strongly reduced. Furthermore, the broad normal-superconductor transition displayed in the magnetoresistivity curves does not allow a very accurate determination of its onset value.

We have to distinguish two distinct sets of simulations: the first for H_{c2} displayed in Fig. 4 and the second of the magnetoresistivity displayed in Figs. 2 and 3. The samples used for measuring H_{c2} and magnetoresistivity have different critical temperatures and consequently we have used different parameters for their simulations.

The parameters used to simulate H_{c2} and IMR are summarized in the Supplementary Table 2. T_c is defined by experiment and critical temperatures T'_{c1}, T'_{c2}, T'_{c3} can be obtained from $\bar{\lambda}_1$, $\bar{\lambda}_2$, $\bar{\lambda}_3$, γ_{12}, γ_{23} and γ_{13} by the expression $T'_{cl} = e^{\mathrm{C}+2}\Omega_{\mathrm{BCS}} e^{-1/\lambda_l}/\pi$, where λ_l is related with $\bar{\lambda}_l$ and γ_{ij} (see in text above) and C is the Euler constant. We expressed all coupling parameters as a function of a single coupling constant λ and T'_{cl} such that the superconducting critical temperature obtained from

experiments is related with λ by $T_c = e^{\mathrm{C}+2}\Omega_{\mathrm{BCS}}e^{-1/\lambda}/\pi$. The absolute values of the coupling constants $\bar{\lambda}_l$ for our compound were not yet determined by experiments. However, according to theoretical study in Ref. [9] the value of the strongest coupling constant for a similar doping levels is ≈ 2, consequently we took $\lambda = 2$.

The set of parameters, $\bar{\lambda}_1$, $\bar{\lambda}_2$, $\bar{\lambda}_3$, $\bar{\gamma}_{12}$, $\bar{\gamma}_{23}$ and $\bar{\gamma}_{13}$ is not unique since, the values of the coupling constants, $\bar{\gamma}_{12}$, $\bar{\gamma}_{23}$and $\bar{\gamma}_{13}$, between different components can be changed arbitrarily such that simulation results of H_{c2} and IMR remain almost unchanged, if we also change the critical temperatures or the parameters $\bar{\lambda}_i$ associated with $\bar{\phi}_i$, where $i \in \{1,2,3\}$. Additionally, the highest $\overline{\lambda_i}$, designated by $\bar{\lambda}_m$ is defined such that $T'_{cm} = T_c$.

Supplementary Figs. 19 and 20 display the simulation results corresponding to the parameters in and fifth and seventh rows and in the first and fourth rows of Supplementary Table 2, respectively. The simulations corresponding to second, third, and sixth rows of the same table are displayed in Figs. 3 and 2.

In the Fermi surface there are electron pockets each on sides of the Brillouin zone, see Supplementary Figure 18. They are eccentric which allows the mixing between harmonic functions which have $s_\pm$-wave and $d_{x^2-y^2}$-wave symmetries in the centers of the pockets, displayed in Supplementary Eqs. (9)-(14), Eqs. (40)-(42) and Eqs. (43)-(45). Note that both electron pockets will compensate each other and will have well defined fourfold symmetry. To take into account the mixing for each component of the order parameter, we set a fix mixing of 10% between the harmonic functions with the same symmetry of the component and the harmonic functions which are allowed to be mixed on the electron pockets, i.e. $a_{i,2}/a_{i,1} = 0.1$ where $i \in \{1,2,3\}$. The absolute values of $a_{i,1}$ and $a_{i,2}$ can be found by the normalization of $\bar{\phi}_i$. We varied this ratio value and found that it only influences very weakly the H_{c2} and magnetoresistivity results.

In the calculation of magnetoresistivity $U_0 = \mathrm{A}\Delta G \frac{\Phi_0}{\mathrm{B}} \xi(T)$ where A is a proportionality factor that was extracted by fitting the simulated to the experimental curves, $\mathrm{A} = 0.01$. The value of $\xi(T) = \xi_0(1 - T/T_c)$ was set to $\xi_0 = 3.53$ nm according to Ref. [2].

Supplementary Note 7. Estimation of the impact of misalignment in H_{c2}

We can assess the impact of misalignment in H_{c2} by calculating the relative deviation of the magnetic critical field, i.e.

$$d(\varphi) = \frac{B_{c2}(\varphi,0) - B_{c2}(0,0)}{B_{c2}(0,0)}. \tag{62}$$

For this calculation we will consider only a single s-wave component since magnetic field cannot the mix the s-wave and the d-wave components. We will present here mathematical expressions that are shown, below, in theoretical description. We can obtain $B_{c2}(\varphi, \theta)$ using Supplementary Eq. (44).

If we only take into account the ϕ_1 component, it becomes:

$$0 = w_{(0,0),1} - B_{c2} w_{(2,0),1} (\phi_1^2). \tag{63}$$

In the case of a s-wave component $\phi_1 = 1$. For simplicity we will drop the index 1 in all the coefficients and functions, and explicitly express the dependency of the variables on the angles, φ and θ, that define the direction of the applied field. The previous equation becomes

$$0 = w_{(0,0),1} - B_{c2} w_{(2,0),1} (\varphi, \theta). \tag{64}$$

Thus,

$$B_{c2} = \frac{w_{(0,0),1}}{w_{(2,0),1}(\varphi,\theta)}, \tag{65}$$

where

$$w_{(2,0),1}(\varphi,\theta) = \langle |\bar{v}_F(\varphi,\theta)|^2 \rangle \sum_{n=0}^{\infty} \frac{(2\pi)^2 T \hbar^2}{2^3 \varepsilon_n^3 \Phi_0}. \tag{66}$$

The relative deviation of B_{c2} becomes:

$$d(\varphi) = \frac{B_{c2}(\varphi,0) - B_{c2}(0,0)}{B_{c2}(0,0)} = \frac{\langle |\bar{v}_F(0,0)|^2 \rangle}{\langle |\bar{v}_F(\varphi,0)|^2 \rangle} - 1. \tag{67}$$

We can find the value of $\bar{v}_F$ using Supplementary Eq. (37), where v_{Fx} and v_{Fy} are given by Supplementary Eqs. (40) and (41) respectively and c_1 and c_2 are given by:

$$c_1 = \left(\frac{\langle v_{Fy}^2 \rangle^2}{\langle v_{F_x}^2 \rangle \langle v_{Fy}^2 \rangle - \langle v_{F_x}^2 v_{Fy}^2 \rangle} \right)^{1/4}, \tag{68}$$

$$c_2 = \left(\frac{\langle v_{Fx}^2 \rangle^2}{\langle v_{F_x}^2 \rangle \langle v_{Fy}^2 \rangle - \langle v_{F_x}^2 v_{Fy}^2 \rangle} \right)^{1/4} \exp\left(i \tan^{-1} \left(\frac{-\langle v_{Fx} v_{Fy} \rangle}{\sqrt{\langle v_{F_x}^2 \rangle \langle v_{Fy}^2 \rangle - \langle v_{F_x}^2 v_{Fy}^2 \rangle}} \right) \right) \tag{69}$$

Using the Fermi velocities and the Fermi surface of the tight-binding model we estimate that a misalignment of 2 degrees induces a $d(2^{o})$=0.033%, which is negligible when compared with the strong anisotropy of 14.4% in H_{c2}.

Supplementary Note 8. Metastable states in measurements of the angle-dependent in-plane magnetoresistivity

The procedure taken for measuring angular dependence of IMR (displayed in all figures to the exception of Supplementary Figure 20) consists in lowering the temperature, with sample is along *a*/*b*-axis (we defined it as $\theta = 0^{\circ}$), until the temperature at which we want to measure to IMR. Then, we rotate the sample along the *c*-axis and measured the IMR for each angle (with a given fixed angle step). While lowering the temperature along the *a*/*b*-axis, we can be trapping the system into a state that is a stable minimum for that direction but not for the other directions for which the IMR is going to be measured when the sample is rotated. To assess the metastability of the system we measured IMR for each desired angle after rotating the sample at 150 K and then lowering temperature until a given value. Supplementary Figure 18 show values of IMR taken using this new methodology and contrasts then with IMR values taken as all the other measurements in this work (as described in the beginning of the section). In this figure, we can observe that both methodologies give similar results, thus, excluding the presence of metastable states that explain the C_2 shape in the angular dependence of the IMR.

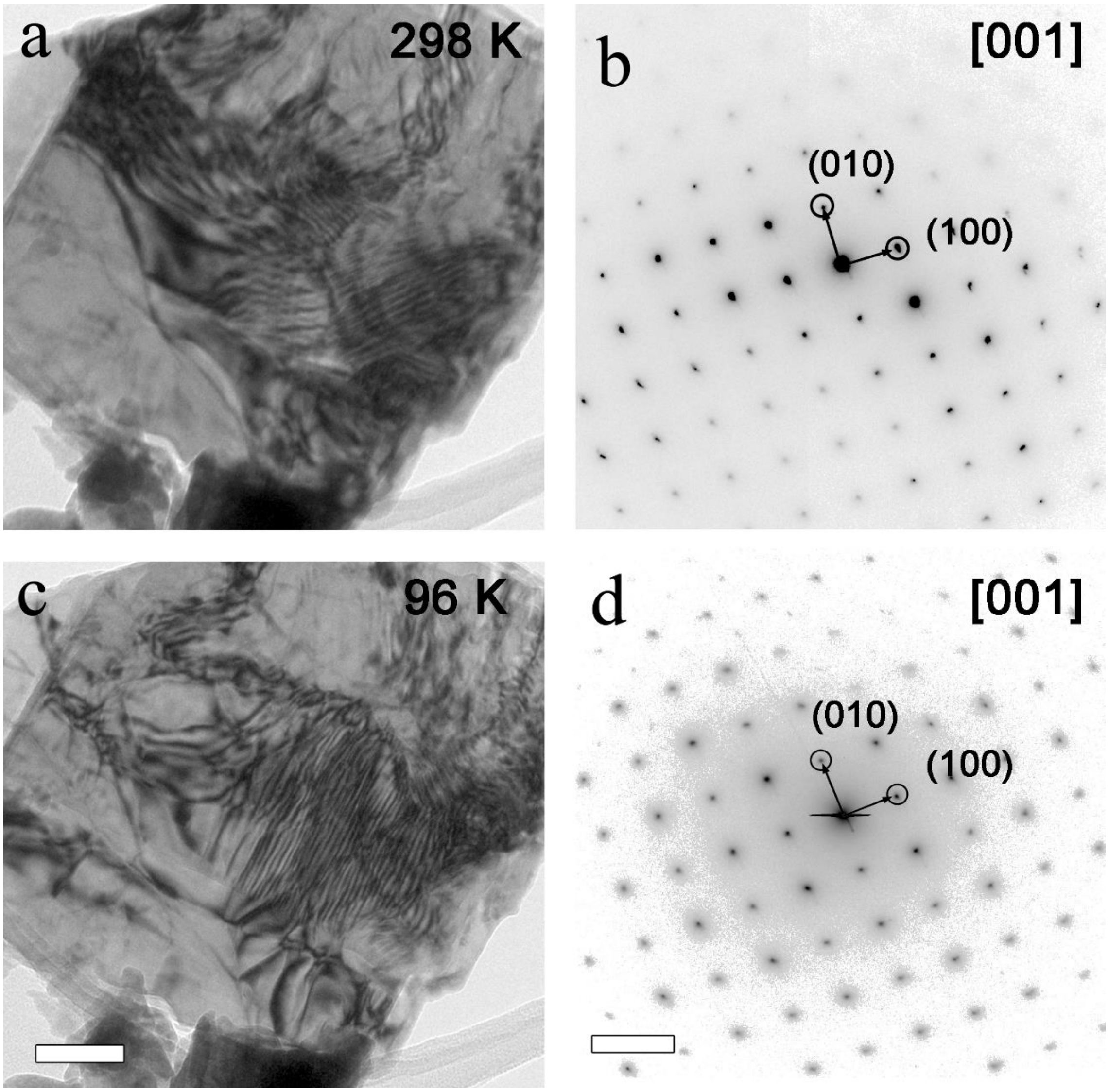

Supplementary Figure 1. Low temperature TEM measurements. (a) TEM image of a crystal in temperature of 298 K and (c) 96 K taken from the [001] direction. The corresponding SAED patterns at the marked positions on the crystal in (b) 298 K and (d) 96 K. Low temperature TEM measurements show the absence of intrinsic twin boundaries at temperature above 96 K, which is consistent with the low temperature XRD analysis on the optimally doped (Ba,K)Fe_2As_2 crystals [1]. Scale bar in (c) and (d) are 100 nm and 5 /nm, respectively.

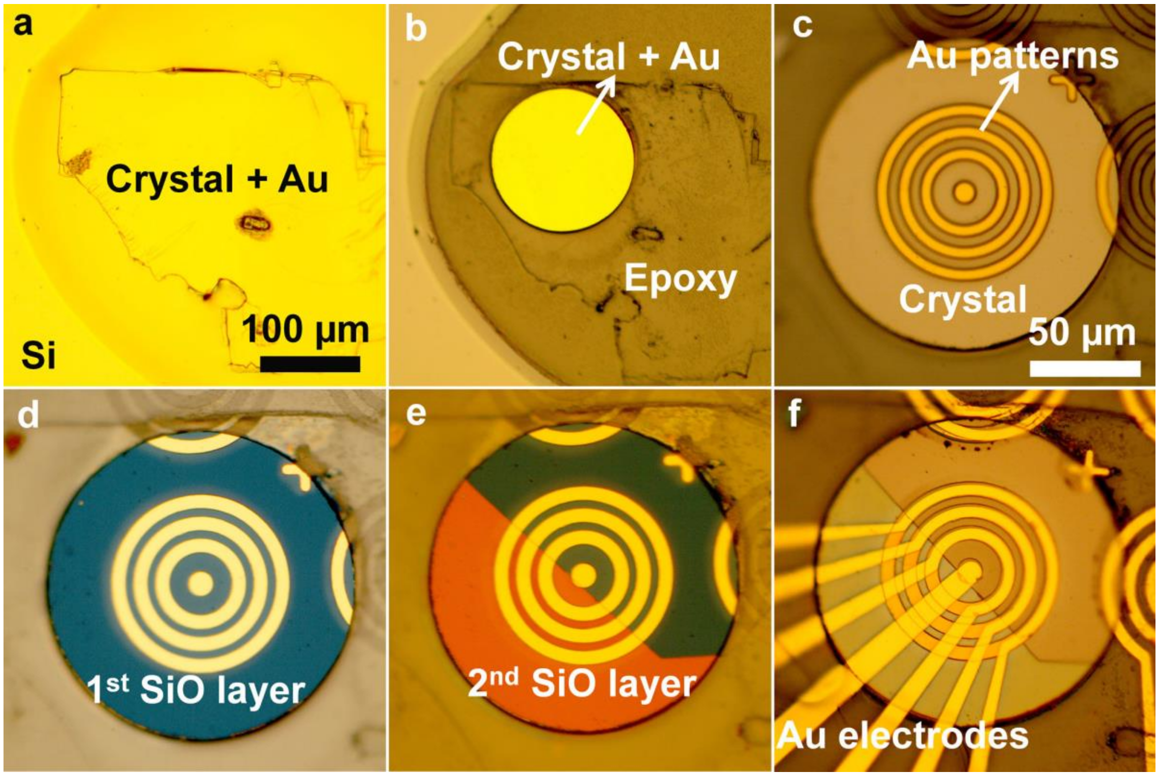

Supplementary Figure 2. Optical photos for the Corbino sample in different processes. Diagram of fabrication process for the Corbino disk device. (a) A cleaved crystal was coated with an approximately 120 nm layer of Au. (b) The flake crystal was fabricated into a disk shape. (c) A set of concentric circles of Au patterns was etched on the *ab*-plane. (d) A 100-nm insulating SiO layer was coated to surround the edges of the mesa. (e) Half of the mesa was covered with a 100-nm insulating SiO layer. (f) The electrodes were formed on the circular mesa by photolithography and argon-ion etching.

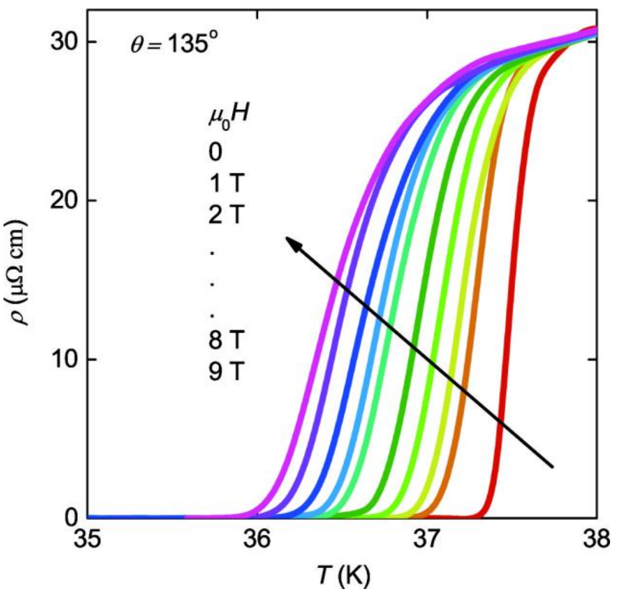

Supplementary Figure 3. Temperature dependence of magnetoresistivity. Temperature dependence of magnetoresistivity for the optimal-doped single crystal $Ba_{0.5}K_{0.5}Fe_2As_2$ under magnetic fields from 0 to 9 T. Here the magnetic fields were applied within the *ab*-plane with angle θ = 135º. A superconducting transition shows an onset temperature of 37.6 K and a zero-*R* temperature of 37.3 K without a magnetic field. This sharp transition suggests that the quality of the single crystal is high, although the transition became wider with increasing magnetic field. The angle θ is defined as that between the magnetic field and the *a*(*b*)-axis of the lattice, as indicated in Fig. 1a. The dependence of ρ_{ab} on θ was measured by rotating the *ab*-plane around the *c*-axis in a fixed magnetic field parallel to the *ab*-plane and at a fixed temperature.

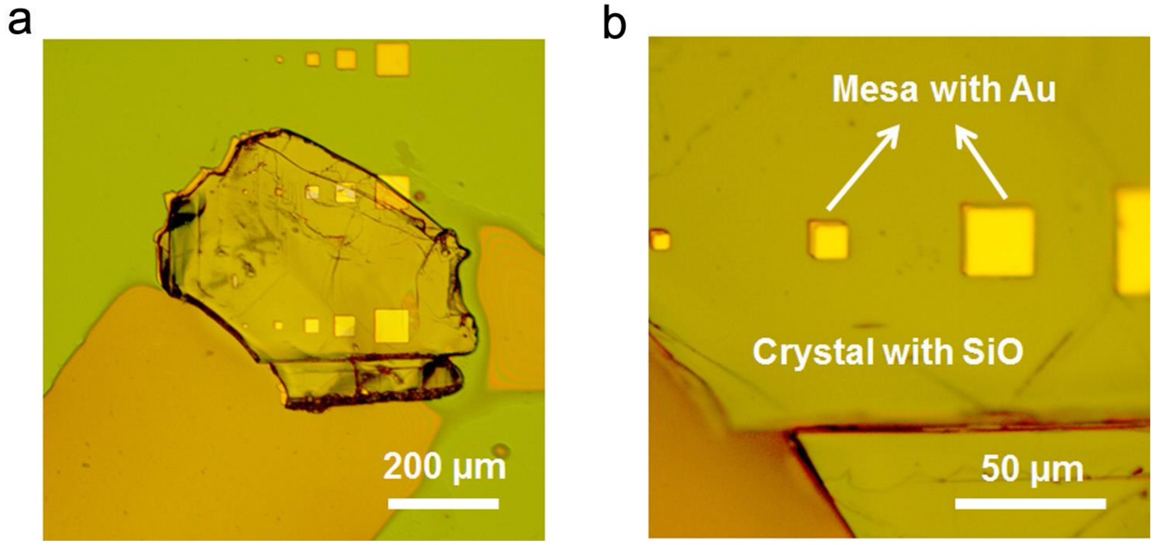

Supplementary Figure 4. Optical photos for mesa sample. Diagram of fabrication process for the mesa device. (a) Mesas were fabricated on a cleaved crystal. (b) Enlarged view of the mesas (10×10, 20×20, and 40×40 μm^2 in area and 1.5 μm in thickness from left to right). The thickness of the base crystal was larger than 20 μm, and the in-plane geometry was considerably larger as few hundred micrometers. Therefore, the resistance of the base crystal was extremely lower than that of the mesa.

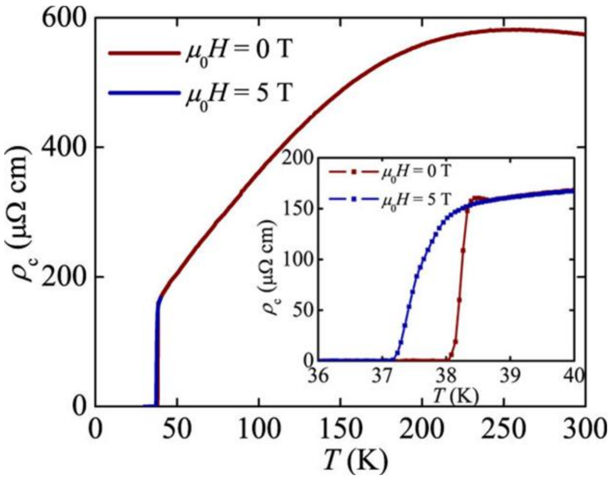

Supplementary Figure 5. Out-of-plane resistivity. Temperature dependence of out-of-plane resistivity ρ_c for $Ba_{0.5}K_{0.5}Fe_2As_2$ in a magnetic field of 5 T. The angle between the field and *a*(*b*)-axis was 45°.

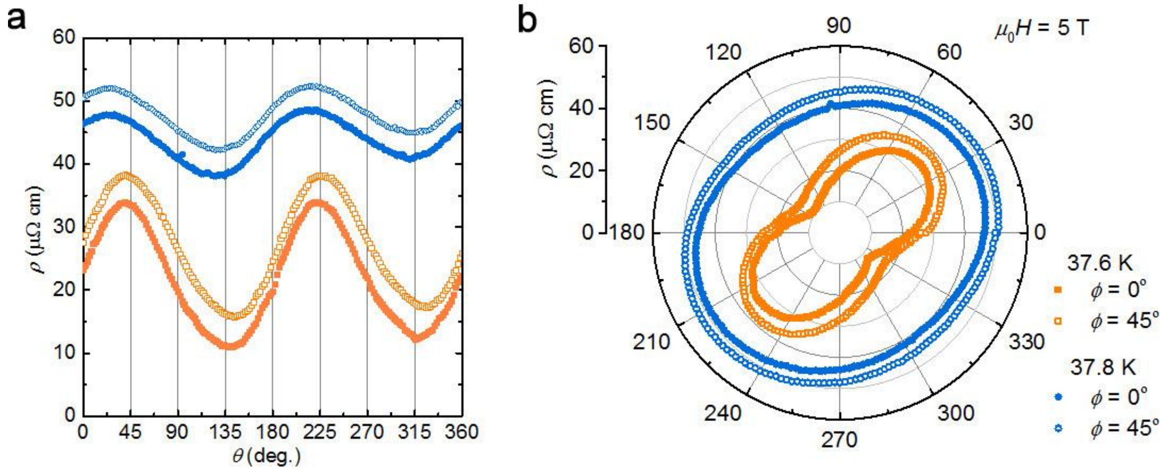

Supplementary Figure 6. Out-of-plane magnetoresistivity under in-plane fields. Angular dependence of out-of-plane magnetoresistivity for $Ba_{0.5}K_{0.5}Fe_2As_2$ in an in-plane magnetic field of 5 T for (a) and the corresponding polar plots for (b). The ρ_c measurements were conducted at initial angles (ϕ) of 0 and 45° between the field and a(b)-axis. As expected, the ρ_c-T curve is slightly different from the ρ_{ab}–T curve. The anisotropy parameter is 2.3 at 300 K, suggesting a quite weak electronic dimensionality of two dimensions. The angle-dependent out-of-plane magnetoresistivity shows an anomaly similar to that observed in the IMR measurements. Supplementary Figure 6 shows the out-of-plane magnetoresistivity at 37.6 and 37.8 K under a magnetic field of 5 T. The ρ_c-θ curves are observed to undergo sinusoidal oscillation with the maximum at the angle where H is parallel to the ΓM direction, comparable to the IMR results. In addition, we tested the dependence of θ on the initial angle. Again, we confirmed that the angular dependence of the magnetoresistivity within the ab-plane truly reflects the in-plane nature.

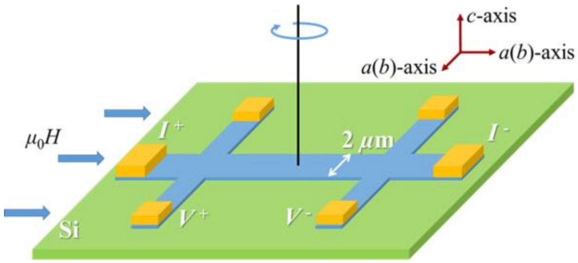

Supplementary Figure 7. Schematic image of a microbridge for pulsed high magnetic experiments. The sample was rotated along the *c*-axis, and the magnetic field was applied within the *ab*-plane.

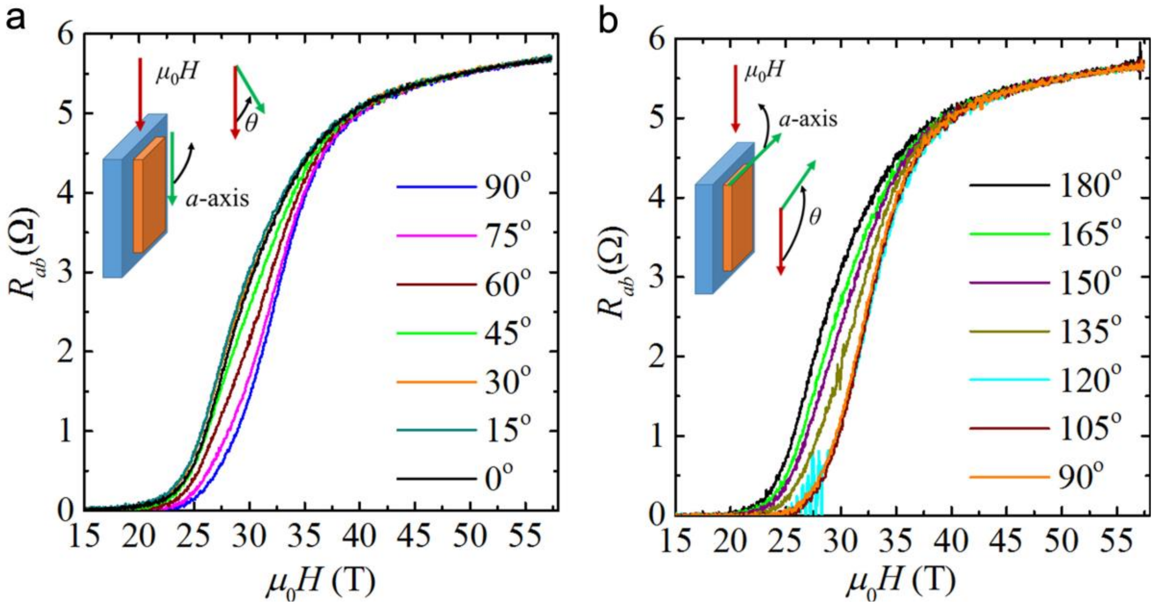

Supplementary Figure 8. In-plane magnetoresistance for $Ba_{0.5}K_{0.5}Fe_2As_2$ under pulsed high magnetic fields up to 57 T. Since the rotation system can provide only 90º rotating angle, we measured the sample within two steps, namely, from 0 to 90º in (a) and 90º-180º in (b), respectively. Here the temperature was fixed at 35 K, and the θ was defined as the angle between the magnetic field and the *a*-axis.

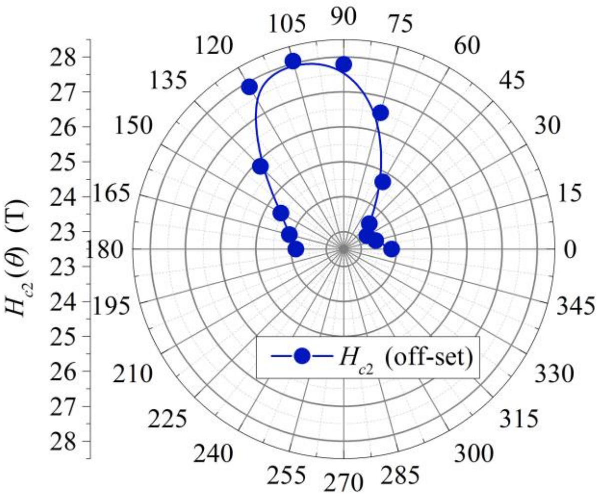

Supplementary Figure 9. Angular dependence of the second magnetic upper critical field. Here the $H_{c2}(\theta)$ data were estimated from the pulsed high magnetic field experiments in Supplementary Figure 9, and are consistent with the static magnetic fields experiments.

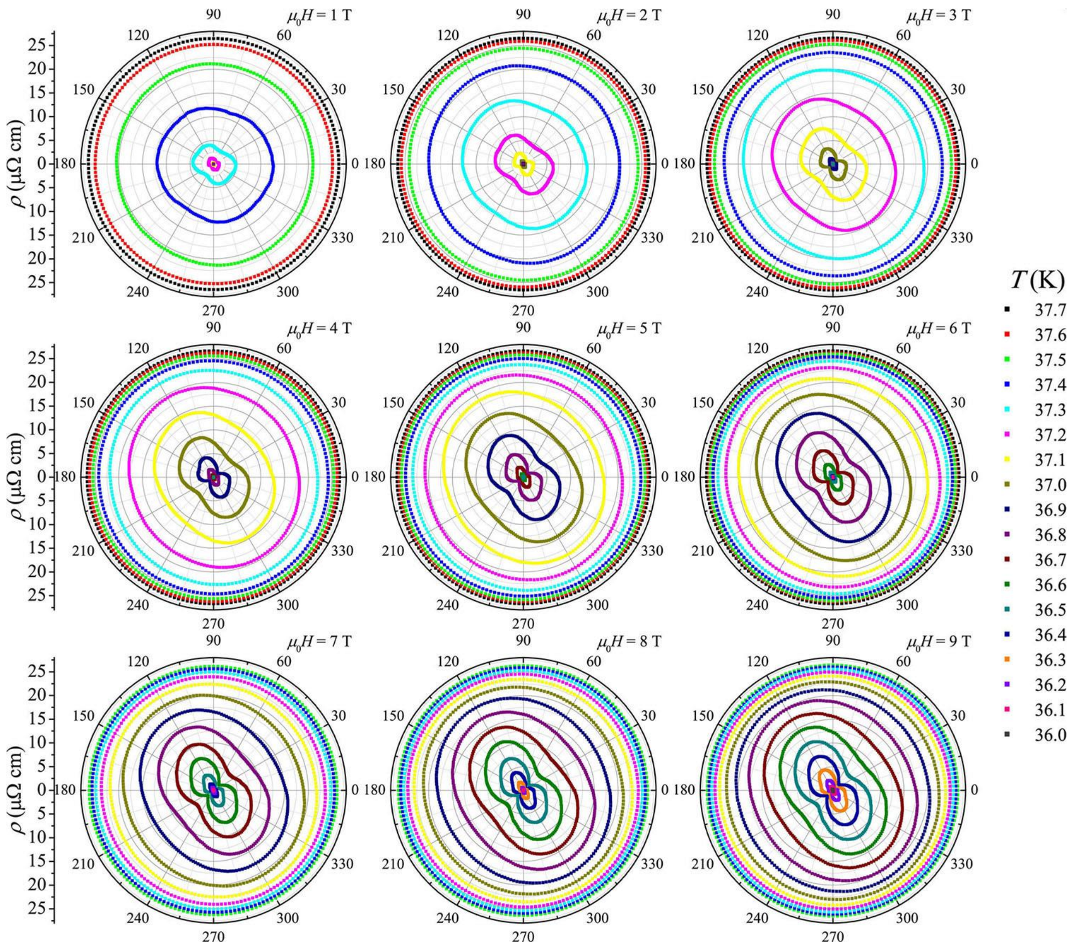

Supplementary Figure 10. Polar plots of in-plane magnetoresistivity. Here, the data are from Fig. 2 for the $Ba_{0.5}K_{0.5}Fe_2As_2$ Corbino sample at various temperatures and magnetic fields. Data for weaker fields show a weakly fourfold symmetric anomaly. The fourfold anomaly gradually transforms to a strong two-fold symmetric anomaly as the magnitude of the applied magnetic field increases.

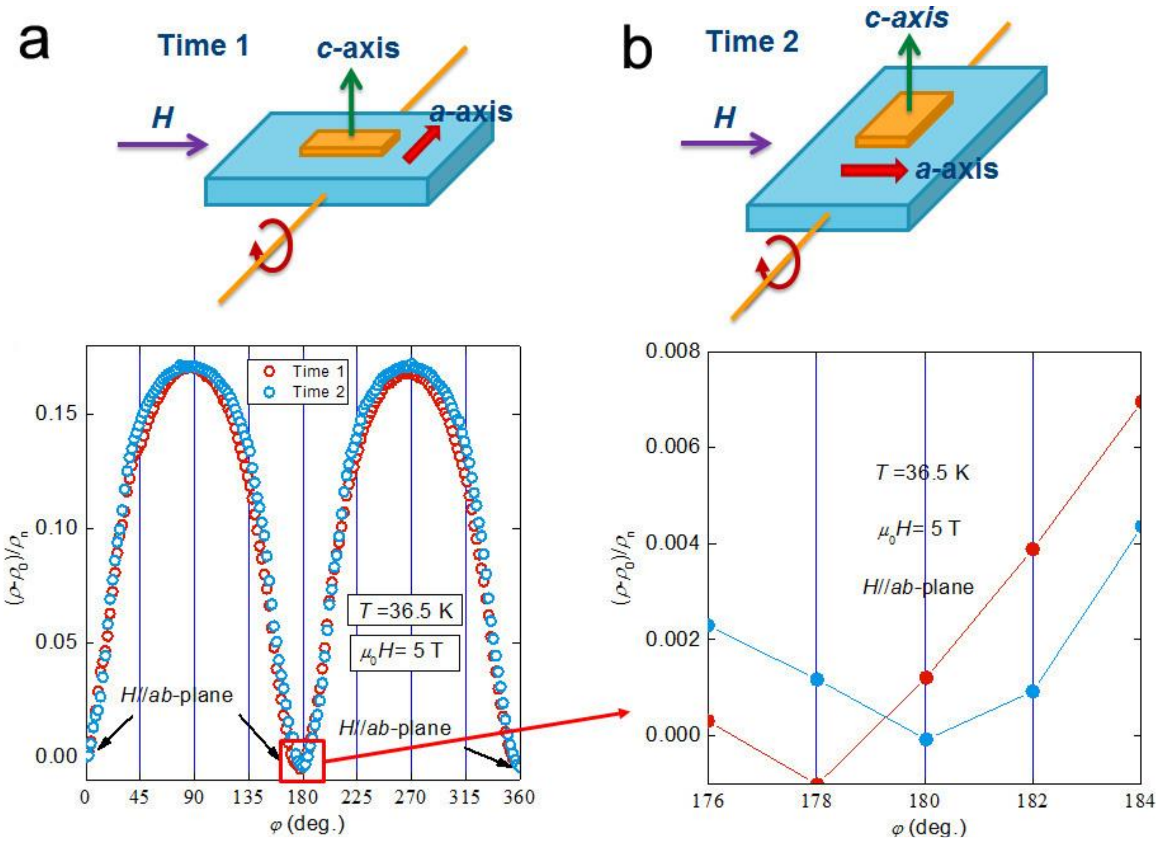

Supplementary Figure 11. Out-of-plane magnetoresistivity measurements. (a) Angular dependence of out-of-plane magnetoresistivity for $Ba_{0.5}K_{0.5}Fe_2As_2$. The magnetic field was rotated from the *a*(*b*)-axis to the *c*-axis. φ is the angle between the *a*(*b*)-axis and *H*. (b) The enlarged view for the field parallel with the *ab*-plane. Up figures are the schematic image for measurement setup. These measurements is to evaluate a possible misalignment between the crystal *c*-axis and the mechanical rotating axis of the sample stage. The maxima appear at exactly 90° and 270° as expected, and the magnetic field is precisely perpendicular to the *ab*-plane, indicating that the *ab*-plane is adequately parallel to the substrate and the sample stage. From the out-of-plane magnetoresistivity data, we estimated that the misalignment is less than ±1°. Even if the angle error is up to ±5°, the influence on the normalized IMR is still very small (e.g., <0.002 at 37.0 K under $\mu_0 H$ = 5 T, compared to the normalized IMR of –0.20 to 0.15).

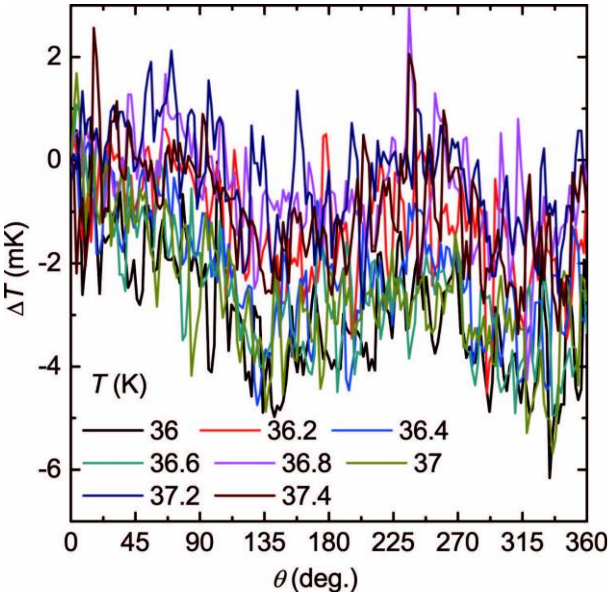

Supplementary Figure 12. Temperature fluctuation. Angular dependence of normalized sample temperature $\Delta T = T - T_{\mathrm{bath}}$, where, T means the temperature of the sample at different anglers, and T_{bath} the bath temperature. The applied field $\mu_0 H$ was 9 T. This experiment is to evaluate the temperature variation of the sample stage in the PPMS, where the angular dependence of temperature is measured at zero-field. The result shows a very weak variation of the sample stage temperature regardless of the angle. The sample temperature difference during rotation is less than 0.008 K. As a result, we can hardly ascribe the large anisotropic IMR data to a temperature problem.

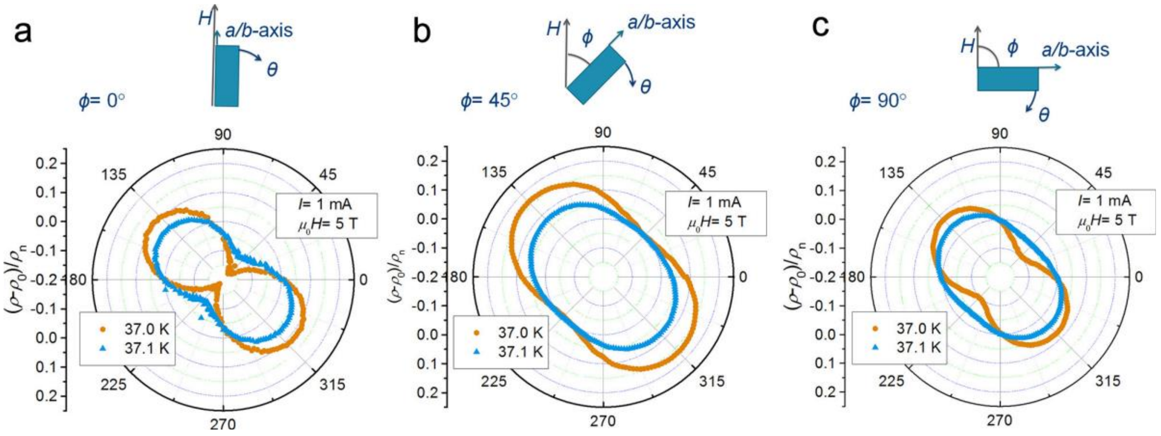

Supplementary Figure 13. Initial anger dependent measurements. Polar plots of normalized IMR with initial angles (ϕ) of (a) 0°, (b) 45°, and (c) 90°. The applied field was $\mu_0 H$ = 5 T, and the sample temperatures were 37.0 and 37.1 K. The polar angle θ is an angle between the magnetic field and a(b)-axis. The maximum (minimum) $\rho_{ab}(\theta)$ was observed along the Fe-Fe direction as well.

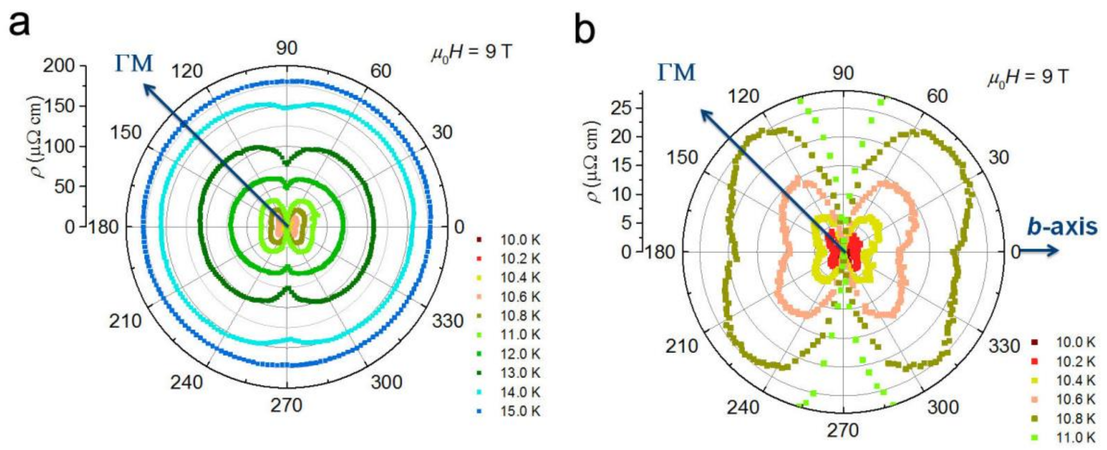

Supplementary Figure 14. Under-doped measurements. (a) Angular dependence of IMR for under-doped single crystal $Ba_{0.75}K_{0.25}Fe_2As_2$ in a magnetic field of 9 T. Here, θ is an angle between the magnetic field and *b*-axis. (b) Angular dependence of the IMR around T_c-onset, where the symmetry can be observed as a mixture four-fold and two-fold with minimum corresponding to *a*- and *b*-axis. Note that the *a*-axis minimum is lower than that of *b*-axis, which is probably owing to the two-fold structural distortion induced nematic state [12]. The tetragonal phase of the compound family is of the $ThCr_2Si_2$-type structure (space group *I*4/*mmm*) and the orthorhombic phase is of the *β*-$SrRh_2As_2$-type structure (*F/mmm*), see Ref. [1]. We note that the *a*- and *b*-axis of the tetragonal lattice structure are rotated 45 degrees from the orthorhombic lattice structure.

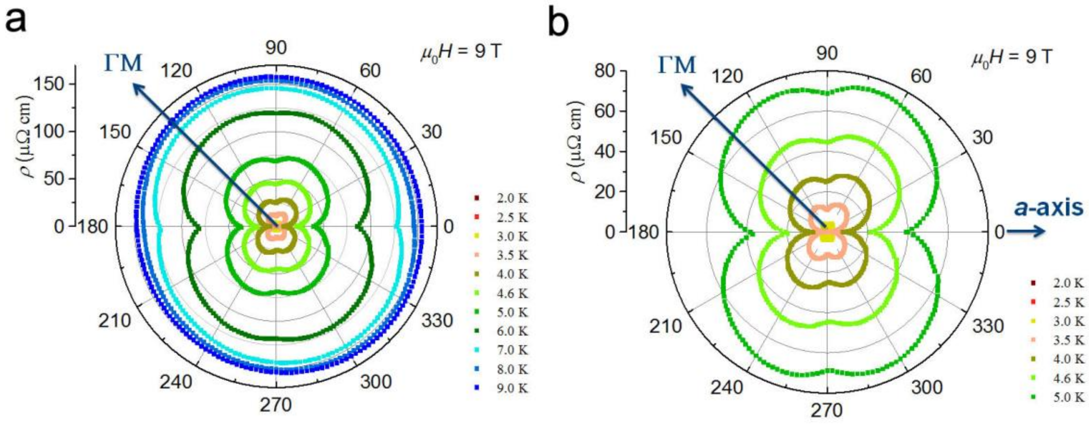

Supplementary Figure 15. **Under-doped measurements for another sample.** (a) Angular dependence of IMR for under-doped single crystal $Ba_{0.8}K_{0.2}Fe_2As_2$ in a magnetic field of 9 T, and (b) the enlarged view of IMR around T_c-onset. Here, θ is an angle between the magnetic field and *a*-axis. The symmetry can be also observed as a mixture four-fold and two-fold as those of $Ba_{0.75}K_{0.25}Fe_2As_2$ sample. The anisotropic behavior between the *a*-axis and *b*-axis may be due to the structural distortion induced nematic state as well.

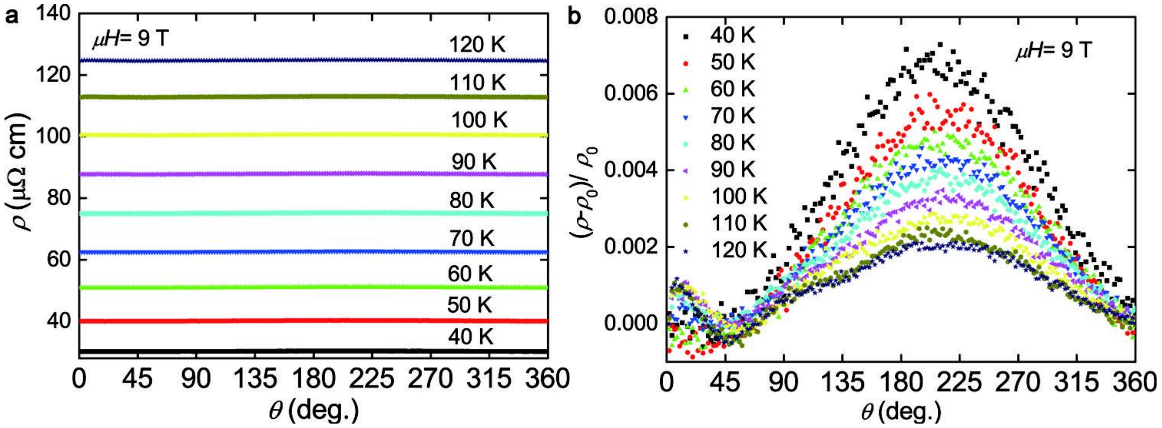

Supplementary Figure 16. **Normal state measurements. Angular** dependence of IMR (a) and normalized IMR (b) in the normal phase for the single crystal $Ba_{0.5}K_{0.5}Fe_2As_2$ in a magnetic field of 9 T, from 40 K to 120 K. The dominant C_1 feature in the angular dependence (b) cannot be attributed either to resistivity or magnetoresistivity angular oscillation because any of them should display invariance under space inversion. The one-fold symmetry of the angular dependence can only be due to drawbacks in the measuring procedure. As a result, the amplitude of the two-fold symmetry oscillation of (magneto) resistivity, signalizing small tetragonal symmetry breaking in the sample due to anisotropy of internal stresses, is expected to be lower than the total amplitude of oscillation displayed in plot (b).

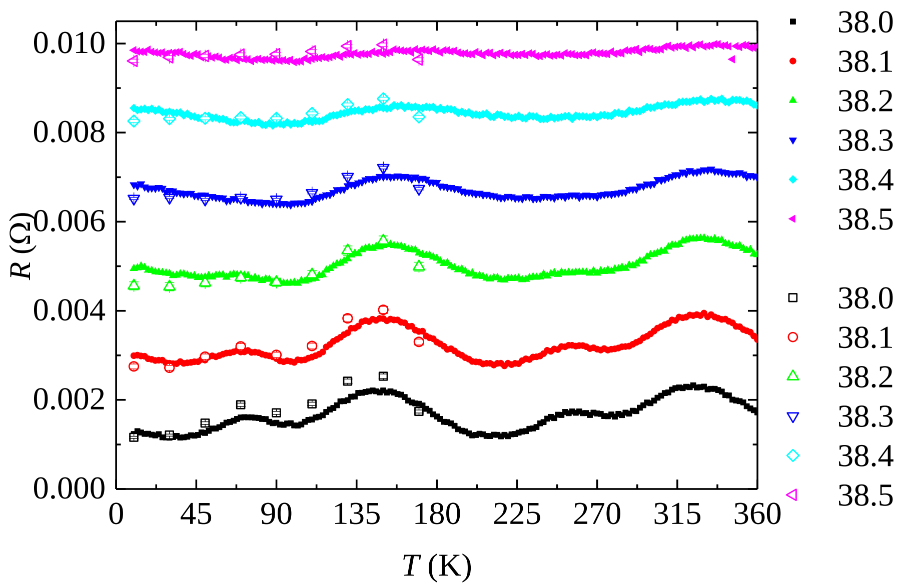

Supplementary Figure 17. IMR measurements after high temperature (150 K) treatment. Angular dependence of IMR, from 38.0 K to 38.5 K, in resistance domain of the normal to superconductor transition after the nucleation of superconductivity. Filled and unfilled symbols correspond to values of IMR measured after the rotation of the sample at the measured temperature or at 150 K temperature.

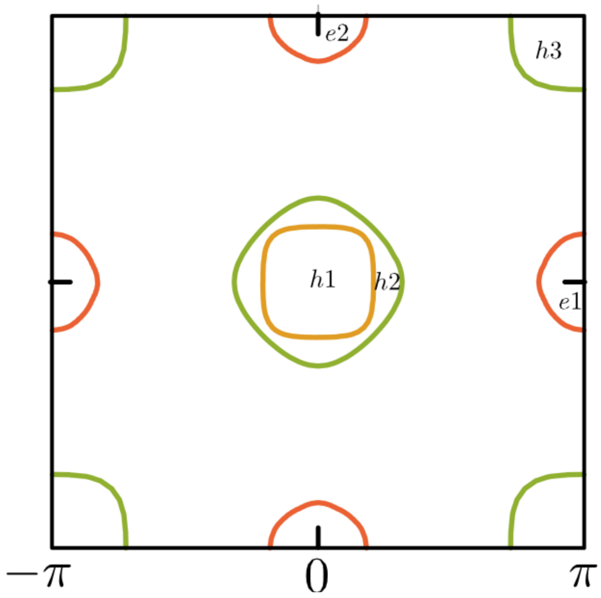

Supplementary Figure 18. Cut of the Fermi surface of $Ba_{0.5}K_{0.5}Fe_2As_2$. The Fermi surface is in the $\boldsymbol{Z} = 0$ plane of the extended Brillouin zone (X- and Y-axis of the conventional lattice are aligned along the Fe-Fe bond directions) corresponding to the $\boldsymbol{Z}$-axis direction of the conventional lattice. Lines in green, yellow and red represent the Fermi surface in distinct bands. The Fermi surface is disconnected and can be subdivided into three hole pockets, $h1$, $h2$ and $h3$ and two electron pockets $e1$ and $e2$, identified inside each pocket the figure.

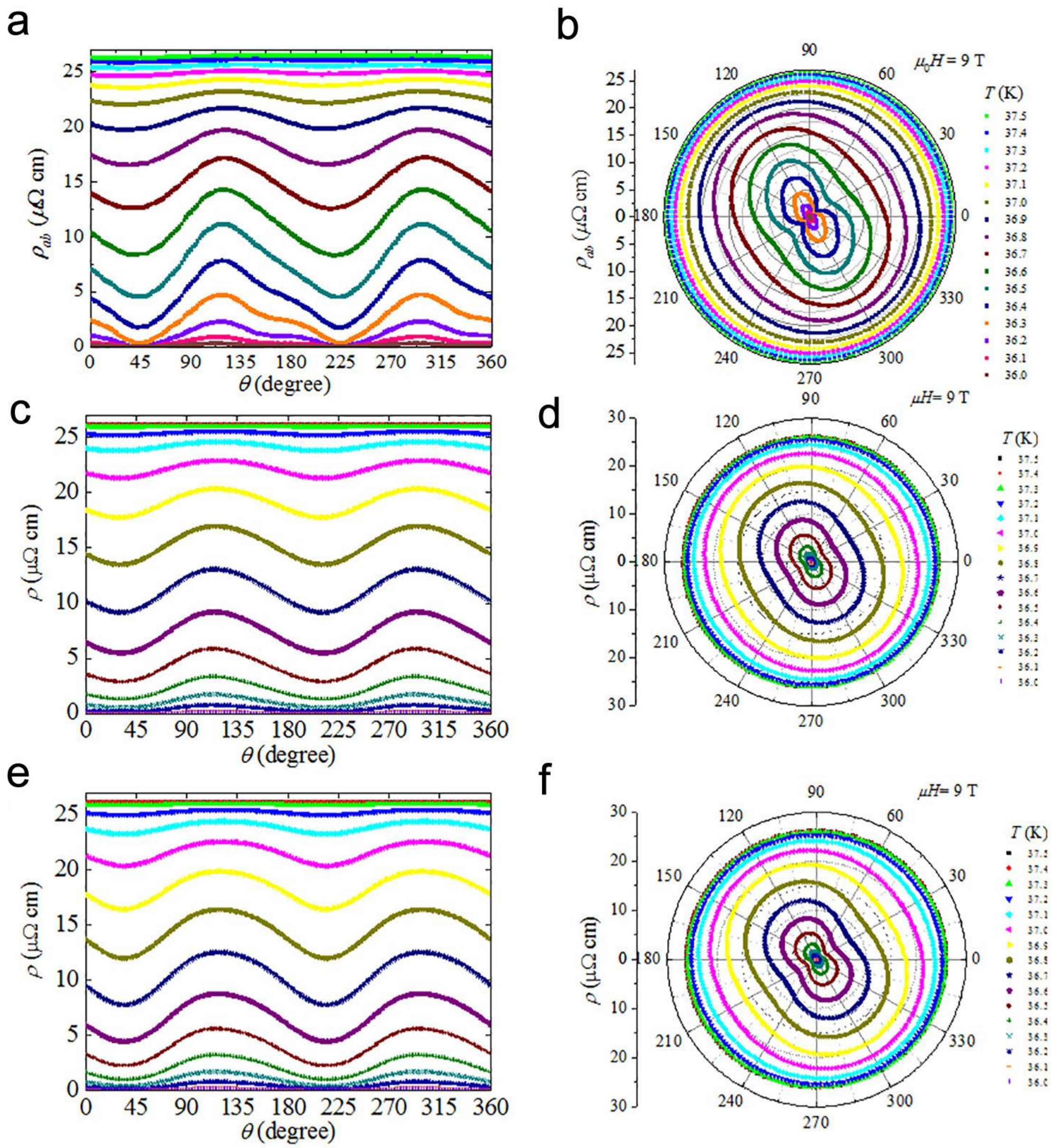

Supplementary Figure 19. **Experimental and theoretical of the angular dependence of the IMR.** Experimental (a) and theoretical (c, e) values of the angular dependence of the IMR, and respective polar plots of IMR experimental (b) and theoretical (d, f) values, at various temperatures for the applied magnetic field of 9 T for which the experimental values were obtained using the Corbino disc measurement configuration. Theoretical values (c, e) of the angular dependence of the IMR, refer to the models where the order parameter has components $s_{\pm}$-wave, $d_{x^2-y^2}$-wave symmetry and an additional d_{xy}-wave and g-wave symmetry component, respectively.

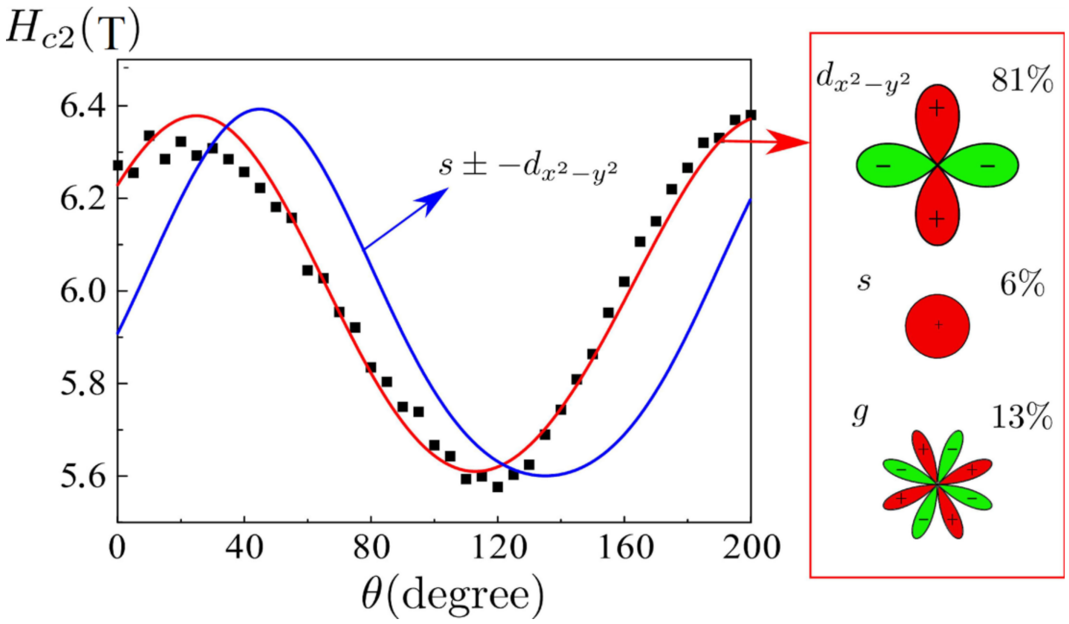

Supplementary Figure 20. Angular dependence of the second magnetic critical field at 38.4 K ($T_c \approx$ 39 K). Here, the data are retrieved from transport experiments (black filled square symbol) in the Corbino disc measurement configuration, from theoretical model with *s*-wave and $d_{x^2-y^2}$-wave symmetries (full blue line) and from theoretical model with $s_{\pm}$-wave, $d_{x^2-y^2}$-wave and *g*-wave symmetries (full red line). The full lines in green and blue correspond to parameter values in the first and fourth rows of Supplementary Table 2. The mixing of the different symmetry components of the order parameters is indicated on the right side panel next to a schematic representation of each component of the order parameter as function of the internal momentum of the Cooper pairs. The indicated percentages correspond to the relative weights (r_1, r_2 and r_3) of the wave function coefficients, $\boldsymbol{\phi}(\mathbf{k}) = \boldsymbol{r_1\phi_s}(\mathbf{k}) + \boldsymbol{r_2\phi}_{\boldsymbol{d_{x^2-y^2}}}(\mathbf{k}) + \boldsymbol{r_3\phi_{d_{xy}}}(\mathbf{k})$. In this schematic red and green indicate positive and negative value of the components, respectively.

Supplementary Table 1: Deviations of the IMR values presented in Supplementary Figure 19 from the average value, i.e. $\mathbf{Dev.} = \mathbf{100}\frac{\mathbf{Max}(\rho(\theta)) - \mathbf{Min}(\rho(\theta))}{\overline{\boldsymbol{\rho}(\boldsymbol{\theta})}}$. Shown symbols correspond to curves in the figure with the same symbols.

Symb.	■	●	▲	▼	◆	◀	▶	⬢	★
T	40 K	50 K	60 K	70 K	80 K	90 K	100 K	110 K	120 K
Dev.	0.79%	0.68%	0.56%	0.48%	0.41%	0.35%	0.31%	0.31%	0.26%
Max.	30.40	40.33	51.06	62.82	75.25	87.99	100.68	113.07	124.86
Min.	30.16	40.06	50.78	62.52	74.94	87.68	100.37	100.37	124.54

Supplementary Table 2: Parameters associated with simulations of H_{c2} and in-plane magnetoresistivity, IMR, (first column-"Simul.") for models with two components, with s±- and $\boldsymbol{d_{x^2-y^2}}$-wave symmetry, and optionally a third component, which can be of g- or $\boldsymbol{d_{xy}}$-wave symmetry (second column-"3rd").

Simul.	3rd	T_c	T'_{c1}	T'_{c2}	T'_{c3}	$\bar{\lambda}_1$	$\bar{\lambda}_2$	$\bar{\lambda}_3$	$\bar{\gamma}_{12}$	$\bar{\gamma}_{23}$	$\bar{\gamma}_{13}$
H_{c2}	g	39	29.92	39	38.22	1.3161	1.9928	1.9204	0.0607	-0.0159	0.0467
H_{c2}	d_{xy}	39	28.78	39	37.81	1.2543	1.9872	1.8853	0.0463	-0.0283	-0.0713
H_{c2}	d_{xy}	39	39	30.45	25.96	1.9907	1.3390	1.1110	0.0464	-0.0284	-0.0714
H_{c2}	none	37.5	29.93	37.5	-	1.3159	1.9924	-	0.0723	-	-
IMR	g	37.5	28.65	37.50	35.10	1.3177	1.9951	1.7533	0.0497	-0.0227	-0.0293
IMR	d_{xy}	37.5	27.72	37.50	35.82	1.2543	1.9871	1.8371	0.0717	-0.0293	-0.0245
IMR	d_{xy}	37.5	37.5	28.89	24.68	1.99	1.3143	1.0989	0.0495	-0.0226	-0.0777

Supplementary References

[2] Avci, S., *et al*. Phase diagram of $Ba_{1-x}K_xFe_2As_2$. *Phys. Rev. B* **85**(18), 184507 (2012).

[3] Li, J. *et al*. Direct observation of the depairing current density in single-crystalline $Ba_{0.5}K_{0.5}Fe_2As_2$ microbridge with nanoscale thickness. *Appl. Phys. Lett.* **103** (6), 062603 (2013).

[4] Kopnin, N. *Theory of nonequilibrium superconductivity*, (Oxford University Press) (2009).

[5] Kita, T. & Arai, M. Ab initio calculations of H_{c2} in type-II superconductors: Basic formalism and model calculations. *Phys. Rev. B* **70** (22), 224522 (2004).

[6] Chubukov, A. V. Pairing mechanism in Fe-based superconductors. *Annu. Rev. Condens. Matter. Phys.* **3**, 57-92 (2012).

[7] Tinkham, M. *Introduction to superconductivity*, 2nd edition (Dover) (2004).

[8] Tinkham, M. Resistive transition of high-temperature superconductors. *Phys. Rev. Lett.* **61** (14), 1658-1661(1988).

[9] Graser, S. *et al*. Spin fluctuations and superconductivity in a three-dimensional tight-binding model for $BaFe_2As_2$. *Phys. Rev. B* **81** (21), 214503 (2010).

[10] Drechsler, S.-L., Rosner, H., Grinenko, V. & Johnston, S. Constraints on the total coupling strength to bosons in iron based superconductors, arXiv:1701.00596

[11] Wang, Z.-S., Luo, H.-Q., Ren, C. D., & Wen, H,-H, Upper critical field, anisotropy, and superconducting properties of $Ba_{1-x}K_xFe_2As_2$ single crystals. *Phys. Rev. B* **78** (14), 140501(R) (2008).

[12] Fernandes, R. M. & Millis, A. J. Nematicity as a probe of superconducting pairing in iron-based superconductors. *Phys. Rev. Lett.* **111**, 127001 (2013).
